# Hybridization has localized effect on genetic variation in closely related pine species

**DOI:** 10.1186/s12870-024-05732-y

**Published:** 2024-10-26

**Authors:** Sebastian Szczepański, Bartosz Łabiszak, Martyna Lasek, Witold Wachowiak

**Affiliations:** 1 Department of Plant Ecology and Environmental Protection, Institute of Environmental Biology, Adam Mickiewicz University in Poznań, Uniwersytetu Poznańskiego 6, 61-614 Poznań, Poland; 2 Institute of Dendrology, Polish Academy of Sciences, Parkowa 5, 62-035 Kórnik, Poland

**Keywords:** Hybrid zones, Introgression, Genetic divergence, Speciation, Interspecific gene flow

## Abstract

**Background:**

Hybridization is a known phenomenon in nature but its genetic impact on populations of parental species remains less understood. We investigated the evolutionary consequences of the interspecific gene flow in several contact zones of closely related pine species. Using a set of genetic markers from both nuclear and organellar genomes, we analyzed four hybrid zones (384 individuals) and a large panel of reference allopatric populations of parental taxa (2104 individuals from 96 stands).

**Results:**

We observed reduced genetic diversity in maternally transmitted mitochondrial genomes of pure pine species and hybrids from contact zones compared to reference allopatric populations. The distribution of *mt*DNA haplotypes followed geographic rather than species boundaries. Additionally, no new haplotypes emerged in the contact zones, instead these zones contained the most common local variants. However, species diverged significantly at nuclear genomes and populations in contact zones exhibited similar or higher genetic diversity compared to the reference stands. There were no signs of admixture in any allopatric population, while clear admixture was evident in the contact zones, indicating that hybridization has a geographically localized effect on the genetic variation of the analyzed pine species.

**Conclusions:**

Our results suggest that hybrid zones act as sinks rather than melting pots of genetic diversity. Hybridization influences sympatric populations but is confined to contact zones. The spectrum of parental species ancestry in hybrids reflects the old evolutionary history of the sympatric populations. These findings also imply that introgression may play a crucial role in the adaptation of hybrids to specific environments.

**Supplementary Information:**

The online version contains supplementary material available at 10.1186/s12870-024-05732-y.

## Background

Understanding the processes that shape the genetic diversity of populations and species, that ultimately are at the core of speciation itself, is fundamental to evolutionary biology [1]. Speciation process is usually long, the reproductive isolation is gradually accumulated and strong barriers are needed to be present for species to form. However, a growing body of evidence in recent years is documenting the speciation with gene flow, which involves the reunion of divergent genomes through hybridization (i.e. polyploid or homoploid hybrid speciation) [2–8]. Hybridization is considered as an important source of novelty in evolution – by adding up the genetic variation on which natural selection operates. In that case hybridization can speed the speciation process by incorporating into genomes of one species adaptive variants from another in a process called adaptive introgression [9].

Hybrid zones, where divergent lineages co-occur and hybridization between them is facilitated, have provided invaluable insights into the genetics of local adaptation, reproductive barriers and speciation [10–14]. Despite estimates showing that 10–30% of animal and plant species hybridize regularly [2], the number of well described classic hybrid zones in plants is surprisingly low, given such a high hybridization potential [15]. Important questions regarding the hybrid zones, especially unimodal hybrid zones (such as hybrid swarms) are still to be addressed by fine scale genetic structure studies. Among these questions are those about the impact of hybridization on the species involved: whether it remains localized within the hybrid zone or extends across the species’ range, potentially leading to the transfer of adaptations and/or genetic swamping of ‘pure’ species [16]. Equally important is also to understand how hybridization influences levels of genetic variation – whether genetic variation is elevated, and whether there are differences between neutral and adaptive variation. Moreover, there is interest in assessing the stability of these zones over space and time, determining whether they are long-lasting or ephemeral. By focusing on these issues, we can unravel the evolutionary significance of hybrid zones and discern the relative importance of hybrid speciation compared to other mechanisms driving the species origin [17–20]. Incomplete reproductive barriers (or the absence of them) are necessary for the occurrence of hybrids in a contact zone of related species. Natural hybridization was documented in a number of forest tree species including among others representatives of the genus *Quercus* [21–24], *Picea* [25–27], *Populus* [28–30] or *Betula* [31–33]. However, interspecific gene exchange may have various evolutionary consequences such us formation of hybrids restricted only to contact zones of parental species [34, 35], introgresants resulting from gene exchange between currently isolated parental taxa [36] or the origin of new hybrid species [37, 38]. There are well documented examples of hybridization in pines (e.g. *P. taeda* x *P. echinata* [39]; *P. montezumae* x *P. pseudostrobus* [40]), that are characterized by weak reproductive isolation and often have overlapping distribution ranges [41–45]. Furthermore, this group of species provide the example of homoploid hybrid speciation that was well documented in the Sikang pine (*Pinus densata*) - an endemic pine species from China and a hybrid of *P. tabulaeformis* and *P. yunnanensis* [46]. Due to potential influence of interspecific gene flow, closely related species within this group and other genera are often described as taxonomically challenging to delineate entities in so-called species complexes [47–49].

Here, we focused on closely related pine species from the *Pinus mugo* complex, specifically dwarf mountain pine (*P. mugo* Turra) and peat-bog pine (*P. uliginosa* Neumann), in areas where they come into contact with Scots pine (*Pinus sylvestris* L.). Despite their relatively recent evolutionary history (the species diverged within the last 5 million years [50]) and close genetic relationships [51], the species have distinctive ecological niches, differ in growth form, some phenotypic features and geographical distribution [48, 52]. Scots pine has the largest distribution, and it is the main forest-forming species in Europe and Asia [53]. Dwarf mountain pine is restricted to subalpine areas in European mountains, where it forms large patches of dense shrubs [54]. Peat-bog pine forms small and restricted populations found only in a few wetlands in mountain regions of Central Europe, mostly in southern Poland. Historical range shifts could affect patterns of the species divergence due to the secondary contact and interspecific gene flow during speciation [50]. However, although natural hybridization between the species was previously described [44, 55, 56], the influence of hybridization on genetic variation at intra- and interspecific level is mostly unknown.

In the presented study we analysed large set of individuals from several contact zones of different species composition and many allopatric reference populations of *Pinus mugo*, *P. uliginosa* and *P. sylvestris* to evaluate the role of hybridization on species genomic divergence. Patterns of genetic variation was assessed at organellar and nuclear genomes to test whether hybrid zones are a source of genetic diversity and if hybridization has influence on genetic variation beyond direct contact zones of the species. The study showed contrasting patterns of divergence at interspecific level at the genomes of different mode of transmission and inheritance. We demonstrate that hybridization has localized effect on genetic variation of the analyzed pine species and introgression, although spatially restricted, may play an important role in local adaptation of hybrids.

## Materials and methods

### The study area

The samples analysed included reference populations of three pine species namely Scots pine (*Pinus sylvestris* L.) and the taxa from the *Pinus mugo* complex (*P. mugo* Turra and *P. uliginosa* Neumann). Outside of those contact zones, in Europe, the ranges of Scots pine (*P. sylvestris*) and dwarf mountain pine (*P. mugo*) are nearly nonoverlapping due to altitudinal isolation. Scots pine typically grows from sea level up to 1,000 m above sea level (asl), whereas dwarf mountain pine is found at elevations between 1,100 and 2,200 m asl in mountain chains of Europe (Fig. S1). *Pinus uliginosa* is known only from a few isolated stands, including those contact zones studied here. Four contact zones of different species compositions from southern Poland were included in the study. Two of them, Błędne Skały in the Stołowe Mountains and Torfowisko pod Zieleńcem in Bystrzyckie Mountains include all three pine species and many intermediate forms considered as hybrids. Population from Błędne Skały is estimated for 7000–8000 years old and it grows on the peaks of Cretaceous sandstone rocks [57]. Torfowisko pod Zieleńcem is the largest of the studied pine contact zones growing on peat bog of the total area of about 232 hectares divided in three smaller sites separated by forest patches and routes [58–60]. In both populations the direct influx of the outside pine pollen is believed to be limited [60]. The other two peat bog stands, Bór na Czerwonem reserve and the area near Czarny Dunajec are located in Nowotarska Valley, circa 25 km north from the Tatra Mountains. Both populations are surrounded by a dense Scots pine forest and they are restricted only to *P. sylvestris*, *P. mugo* and they putative hybrids.

### Sampling and DNA extraction

Samples were derived from 96 distinct geographical locations of the reference pure species including *Pinus sylvestris* (66 populations), *P. mugo* (27) and *P. uliginosa* (3). Samples from contact zones of the species were collected from four above-mentioned stands including Bór na Czerwonem reserve, Błędne Skały, Torfowisko pod Zieleńcem and Czarny Dunajec (Table S1). In total, 384 individuals from hybrid zones and 2104 individuals from reference stands were studied. Samples from Poland were collected in years 2021–2023, although some were already available from previous research [61, 62]. The collection and formal identification of the plant material was conducted by the authors of the study and Krystyna Boratyńska and Adam Boratyński. The sampling was conducted based on permissions from Ministry of Climate and Environment (DOP-WPN.61.116.2021.MGr; DOP-WOPPN.61.35.2022.WH) and Polish State Forests (ZG.7021.2.2021). Total DNA was extracted from fresh needles using the Genomic Mini AX Plant extraction kit (A&A Biotechnology, Poland). The concentration of DNA was assessed using the Qubit 4 fluorometer with the Broad Range (BR) Assay Kit and samples were diluted to a working concentration of 40 ng/µl.

### Group assignments of samples from the contact zones

Each individual from contact zones was initially assigned based on its morphology as pure *P. sylvestris*, *P. mugo and P. uliginosa* or a hybrid group. All the samples from the contact zones of the species were genotyped at SSR nuclear markers and diagnostic *trn*L-*trn*F region of the chloroplast genome (see methods below). *LEA* R package [63] was used to cluster samples into pure species and hybrid groups. Based on the individuals’ morphology and genetic data assessments, the final set of samples for each hybrid zone and taxonomic groups included 17–36 individuals that were used for further genetic analyses based on molecular data from both nuclear and mitochondrial genomes.

### Organellar markers

Analysis of mitochondrial DNA, which is inherited in pines in maternal line and transmitted by seeds at short geographical areas as compared to pollen mediated markers, were used to explore the diversity, phylogenetic patterns and possible sources of Polish contact zones of the species. The reference 22 populations of *P. sylvestris* and 16 populations of *P. mugo* from the Eurasian species range were included only for this purpose (Table S1). Polymorphisms at selected *mt*DNA regions were scored using SNaPshot multiplex SNP genotyping method as described in Szczepański et al. [67]. Briefly, PCRs of preselected *mt*DNA markers were carried out in two multiplex reactions containing *PR5*, *PR7*, *PR15*, *PR19*, *PR20*, *PR21*, *PR24* and *PR25*, *PR29*, *PR30*, *PR31*, *PR32*, *nad*1 DNA regions, respectively [58, 64, 65]. Furthermore, the multiplex two contained the species diagnostic fragment of *trn*L-*trn*F intergenic region of the chloroplast DNA that distinguishes paternally transmitted chloroplast genome of the species [66]. There are two possible variants in this locus - variant A is characteristic for pines from *Pinus mugo* complex (*P. mugo*, *P. uliginosa*) and the variant C is observed in *Pinus sylvestris.* The reaction mixture for both multiplexes contained 1 µl of Solis Biodyne HOT FIREPol^®^ Blend Master Mix (5x) polymerase, 0.5 µl of primer mix (5µM of each primer), 3 µl of Milli-Q water and 1 µl of sample DNA (40ng/µl). PCRs included the following steps: initial denaturation (95 °C, 15 min), 32 cycles of denaturation (95 °C, 30 s), annealing (57 °C, 1:30 min), extension (72 °C, 1:30 min) and final extension (72 °C, 10 min). Following the SNaPshot SNP genotyping method the products of the PCR sequencing reaction were purified with exonuclease 1 and phosphatase and the SNaPshot reaction was performed according to the protocol [67]. Results were viewed in Peak Scanner™ Software v1.0 and the polymorphisms at each genotyped region was used to build concatenated sequence of each haplotype.

### Nuclear single sequence repeats (*n*SSR)

Neutral variation at biparentally inherited nuclear genome was genotyped at a set of 14 simple sequence repeats (SSRs) markers in three multiplex reactions as described by Żukowska & Wachowiak, 2017 [67]. The multiplexes comprised the following markers: (I) *psyl42*,* psyl25*, *psyl2*,* psyl18*,* psyl57*,* psyl36* and *psyl44* (II) *ptTX4011*,* ptTX3025*,* ptTX2146 and ptTX4001* (III) *ptTX8446*,* psyl17* and *pTctg4363*. Each PCR was performed in a total volume of 10 µl including 5 µl of Qiagen Multiplex Master Mix, 1 µl of Q-Solution, 0.2 µl of primer mix, 1.8 µl of water, and 2 µl of DNA template. The fluorescently labelled PCR products, along with the GeneScan™ 500 LIZ™ internal size standard (ThermoFisher Scientific, USA), were separated on the Applied Biosystems^®^ 3130xl Genetic Analyzer (Thermo Fisher Scientific, USA). The allele sizes (length of the fragments) were determined using the GeneMapper™ software v. 4.0 (Thermo Fisher Scientific, USA).

### Mitochondrial DNA analysis

Composition and distribution of mitochondrial DNA haplotypes were analysed to assess the genetic relationships between species and phylogeographic structure of the reference stands and hybrid zones populations. Network of *mt*DNA haplotype was analysed using network Median Joining approach [68] and the PopART 1.7 software [69]. Geographical distribution of haplotypes was presented in R, using packages *tidyverse* [70], *raster* [71], *rgdal* [72] and *ggplot2* [73]. The genetic diversity at *mt*DNA between the reference and hybrid populations was measured by effective number of haplotypes (N_e_), haplotype richness (R_h_) and genetic diversity (H_e_) in Haplotype Analysis software [74] and DnaSP 6 [75]. The haplotype richness was adjusted to the size of the population with the lowest number of samples (S_G3, *N* = 8). All the results were visualised in R using *ggplot2* package. The genetic relationships between individuals and populations were analysed using the Principal Component Analysis (PCA) based on haplotype frequencies calculated in Haplotype Analysis and the R packages *factoextra* [76], *FactoMineR* [77] and *ggplot2*.

### Nuclear DNA analysis

Genetic divergence between species and populations was calculated based on the basic genetic statistics at *n*SSR loci including number of alleles, allelic richness and heterozygosity using *adegenet* R package [78, 79]. Genetic relationships based on Nei genetic distance [80] calculated at individual and population level were assessed using Principal Component Analysis (PCA). R packages *adegenet* and *vegan* [81] were used to perform the analysis and *ggplot2* to visualize the results. Admixture in contact zones and reference populations was tested based on analysis in *LEA* R package using the function *snmf*() to estimate individual admixture coefficients from the genotypic matrix, compute least-squares estimates of ancestry proportions and ancestral allelic frequencies [63, 82, 83]. We tested the number of clusters (K) from 1 to 15 and the results were visualised in *pophelper* R package [84]. Then, to test for signatures of historical gene flow at intra and interspecific level we calculated F_ST_ between all the populations in *hierfstat* R package [85] and visualised the results as a correlation plot with *corrplot* package [86]. Finally, to check potential population size changes in the contact zones of the species, we used the TPM (two phase model) test implemented in Bottleneck 1.2.02 [87, 88]. Effective population size was estimated using the molecular co-ancestry method of Nomura (2008) [89], as implemented in NeEstimator v2.1 [90].

## Results

### Organellar DNA diversity

**Fig. 1:**
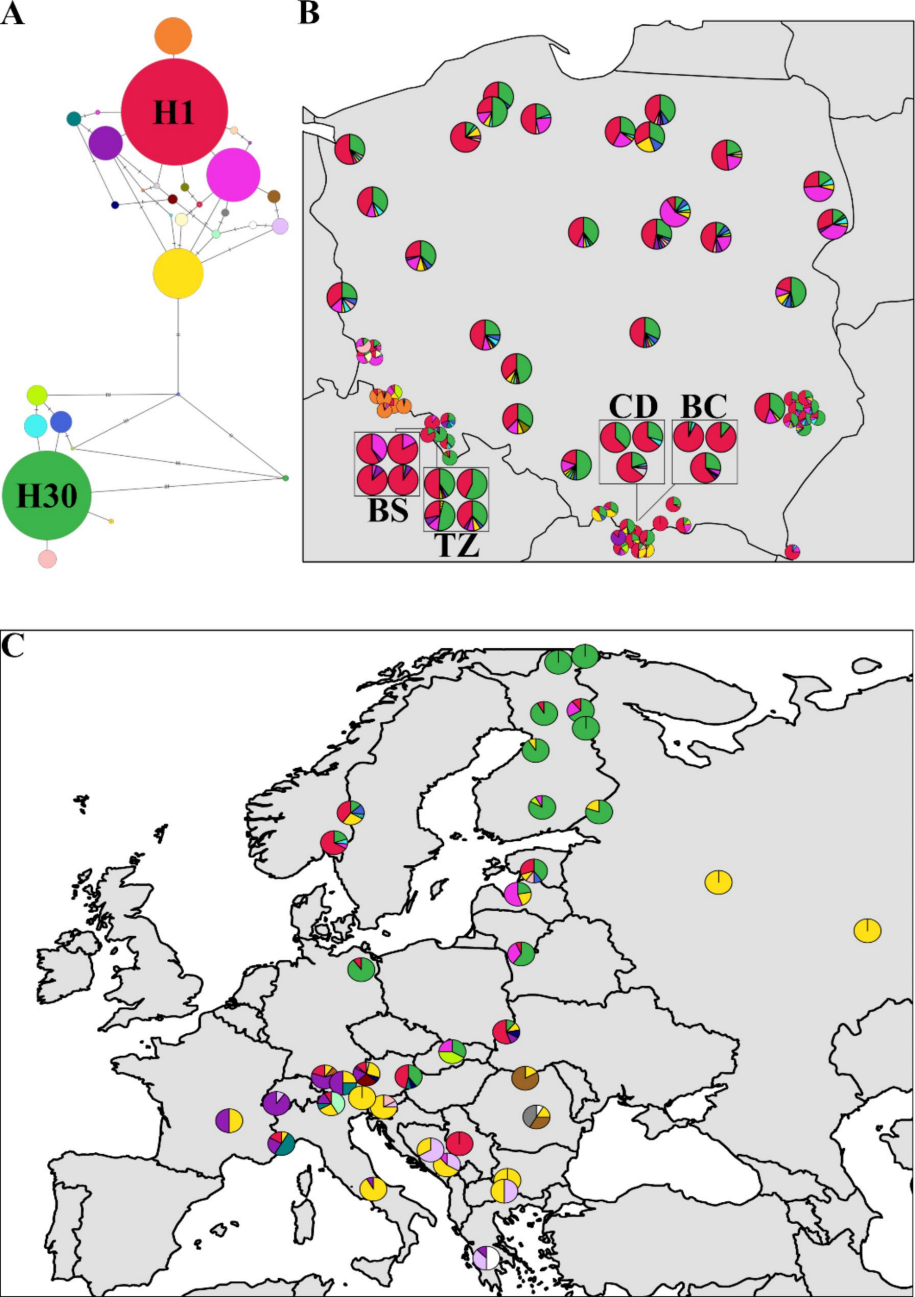
Network of *mt*DNA haplotypes detected (**A**), their distribution in Polish reference and contact zones populations of the species (**B**) and other allopatric European locations (**C**)

**Fig. 2:**
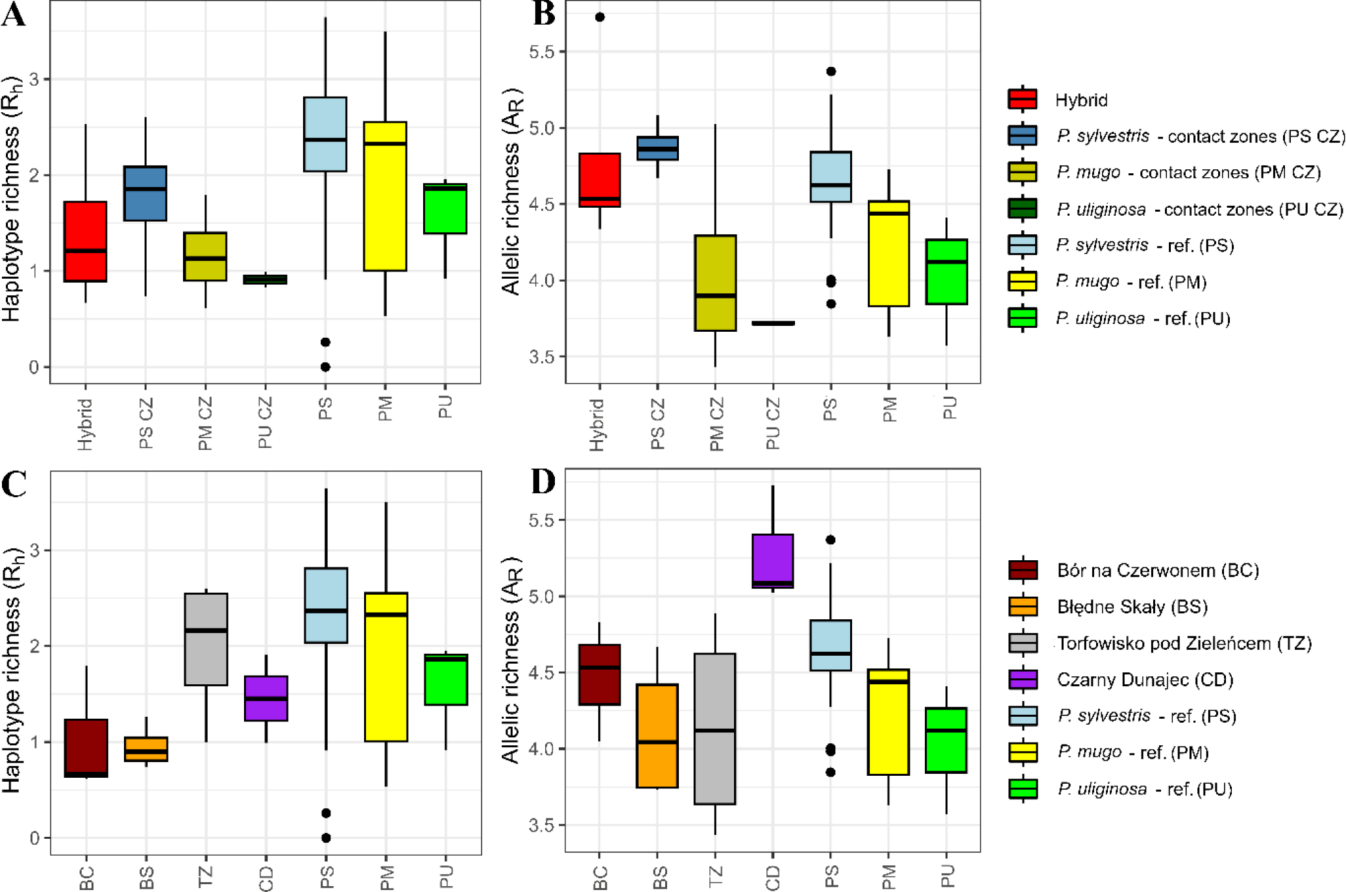
Genetic diversity of selected pine populations. **A**, **C** - mitochondrial haplotype richness (R_h_) estimated for studied groups of populations. **B**, **D** - nuclear allelic richness (A_R_) estimated for studied groups of populations

Mitochondrial DNA markers were successfully genotyped in 2601 samples providing 31 unique haplotypes in 110 populations (Table S2). The haplotypes were divided into two main genetic groups (Fig. 1A). The most common haplotype (H1) was found in 973 individuals in 90 populations. It was most common in *P. sylvestris*, but it was also found in the majority of *P. mugo* populations, two allopatric stands of *P. uliginosa* (WGB and WLB) and it was the most frequent haplotype in individuals from most contact zones of the species. Two most frequent haplotypes (H1 and H30) were found in the majority of trees sampled in this study (~ 63.4%). Several singleton haplotypes (H7, H14, H16, H20 and H27) were found in *P. mugo* and *P. sylvestris* populations. One private haplotype (H3) of high frequency (22.3%) was the common mitotype in dwarf mountain pine stands in Karkonosze Mountains. In general, individuals from hybrid zones shared haplotypes at similar frequencies as found in reference populations of the species and no unique haplotypes were found there. However, most individuals in hybrid zone of the Torfowisko pod Zieleńcem had haplotype H30, the second most frequent one in Poland and dominant in two neighbour reference populations of *P. sylvestris* from Międzylesie (S_Md), *P. uliginosa* population from Wielkie Torfowisko Batorowskie (BAT) and the one fixed in several Finnish Scots pine populations (Fig. 1B, C). The only private haplotype in hybrid zones was H2 which differs from H1 only by one mutation in *nad*1 region. It was only detected in three Scots pine individuals from Czarny Dunajec contact zone (CDS). This rare variant of *nad*1 is characteristic for another species of the *Pinus mugo* complex, *P. uncinata*, and was reported in its Iberian populations [61, 67]. The detected similarity would rather result from close genetic relations in the species complex, rather than hybridization, since the populations are significantly geographically isolated from each other. In our data we found this variant in other rare haplotypes only in 5 samples – in *P. mugo* population M11 from German Alps, in *P. sylvestris* mountain population from Narożnik, Stołowe Mountains and hybrids from Torfowisko pod Zieleńcem (TZH). No species diagnostic mitotypes were found and their distribution followed geographical rather than species boundaries (Fig. 1B, C). Hybrid zone populations showed generally lower number of haplotypes as found in reference populations and the median of haplotype richness of different hybrid classes was lower than any of the parental species groups (Fig. 2A). Similarly, haplotype richness of each hybrid zone was lower as compared to reference populations (Fig. 2C). The exception was the Torfowisko pod Zieleńcem (TZ) peat bog that has a slightly higher R_h_ than *Pinus uliginosa* (Table S3, Fig. 2C). The principal component analysis based on the haplotype frequencies confirmed that there was no clear species division as allopatric populations of all parental species were mixed together (Fig. S2A). However, the defined groups of pure species and hybrids from a specific contact zone were clustered with each other (Fig. S2B).

The reference Scots pine populations and individuals from contact zones were fixed for variant C and the taxa from the *P. mugo* complex were fixed for variant A of the *trn*L-*trn*F diagnostic intergenic region of the chloroplast genome. Both variants were present in individuals from hybrid groups and usually the presence of corresponding variant matched the highest proportion of ancestry of the individual parental species.

### Nuclear DNA diversity

High quality scores were obtained for all 14 *n*DNA markers genotyped in 2073 individuals (Table S4). As a measure of genetic diversity, we chose the allelic richness (A_R_) as a corresponding parameter to the haplotype richness (R_h_) at mitochondrial markers. In contrast to *mt*DNA results, populations tagged as hybrids had higher median allelic richness than *P. mugo* and *P. uliginosa* populations from hybrid zones and allopatric locations. The only groups with higher median allelic richness were *P. sylvestris* from contact zones and reference populations (Fig. 2B). When all individuals from each contact zone were grouped (BC, BS, TZ and CD), the median A_R_ was lowest in BS and TZ hybrid zones, lower than allopatric populations, although BC and CD had one of the highest median A_R_ (Fig. 2D). The calculated parameters of the genetic diversity of each studied population were also presented as bar plots to simplify the interpretation of the results (Fig. S3).

**Fig. 3:**
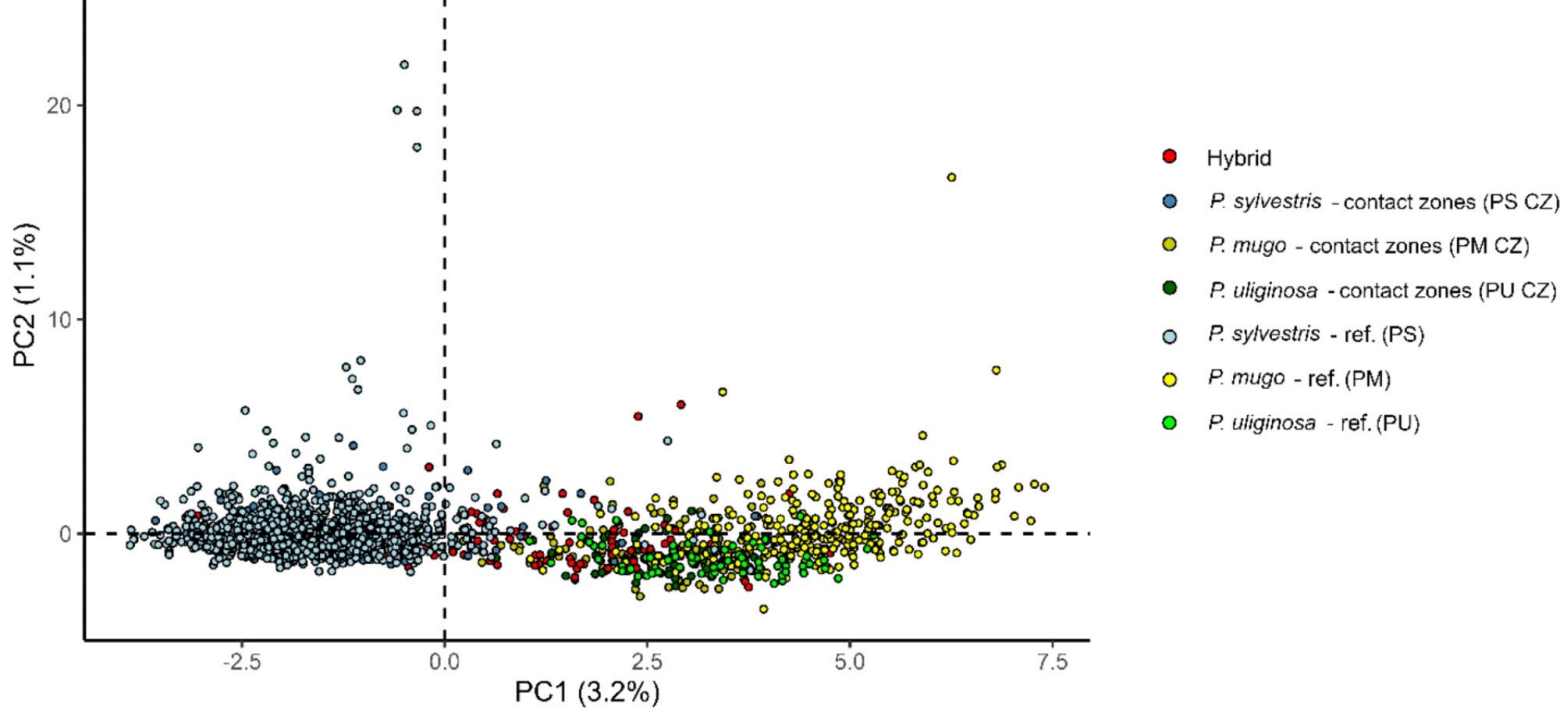
Principal Component Analysis (PCA) derived from nuclear microsatellite data (*n*SSR)

**Fig. 4:**
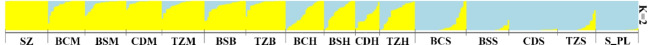
Results of the individual ancestry coefficients analysis for K = 2 derived from nuclear microsatellite data (*n*SSR), for all hybrid zones analyzed and selected reference populations of pure species. Individuals clustered to *P. sylvestris* genetic group are marked in blue, and to *P. mugo* populations in yellow

**Fig. 5:**
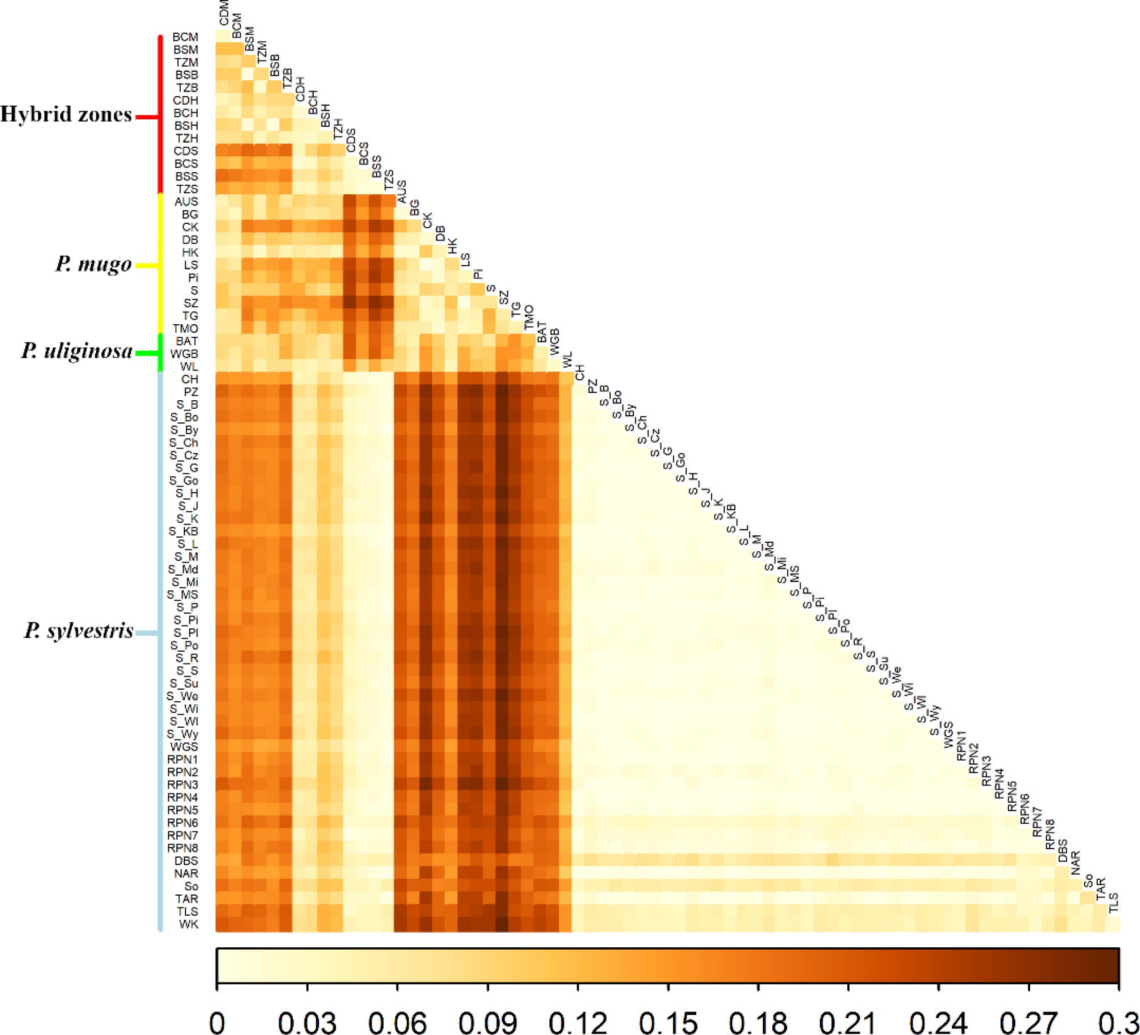
Pairwise F_ST_ values between all the studied populations, derived from nuclear microsatellite data (*n*SSR)

Clear species division between *P. sylvestris* and *P. mugo* complex was observed in Principal Component Analysis at the individual level (Fig. 3) and populations defined to pure species and hybrid classes (Fig. S4). Individual ancestry coefficients analysis indicated the presence of two genetic clusters (K = 2, Fig. S5) corresponding to pure Scots pine and the taxa from the *Pinus mugo* complex (Fig. 4, Fig. S6). Additional results of the analysis for higher K (K = 3 to K = 7) are available in Supplementary Materials (Fig. S7). Evidence of genetic admixture was found only in contact zones populations indicating the most mixed ancestry in the samples grouped as hybrids. The biggest genetic differentiation was observed between allopatric populations of *P. sylvestris* and *P. mugo* (F_ST_ >0.29). Intraspecific variation was lowest in Scots pine populations except some isolated mountain populations of the species. F_ST_ between *P. mugo* reference populations was variable, but higher than for *P. sylvestris* populations and genetic variation of hybrids groups reflected they mixed ancestry as compared to parental species (Fig. 5). Bottleneck analysis did not indicate different patterns of population size changes than in allopatric populations (Table S4). The smallest effective population size was observed for the most of isolated mountain populations of Scots pine and hybrid zones showed similarly large N_E_ as compared to most allopatric populations of the species (Table S4).

## Discussion

Although hybridization is a common phenomenon in many species [91–94], its genetic and evolutionary consequences at intra- and interspecific level are unknown for many, especially non-model plants organisms. Here we focused on a complex of pine species that are known to form contact zones in some relict mountain populations from postglacial periods [44, 55, 95–99]. The hybrid zones can be called “mosaic hybrid zones” in which the transition between the species ranges is not smooth but patchy [1]. Similar sympatric populations were described for example in forest trees [100–102], perennials [103, 104] and some animals [105–107]. Our data shows that those stands contain individuals of pure species and hybrids of mixed proportion of parental genetic backgrounds. That result indicates that hybridization is a repeatable process despite distinct species composition and different environments of such populations. In the analyzed stands fertile hybrids can likely cross with each other and backcross with parental species producing a largely continuous fraction of introgressed genotypes. However, our data provide no evidence that hybridization may affect genetic variation of the investigated species beyond their sympatric populations. We found no evidence of mixed ancestry in any pure species populations and they showed high divergence at pollen mediated markers of the nuclear genome. Even though hybridization could play a role during the speciation of the taxa [50, 51], there is no evidence of more recent interspecific gene flow between allopatric populations of this wind pollinated and highly outcrossing species, which were clustered as separate genetic groups at the population and individual trees level.

### Drivers of mitochondrial variation in the hybrid zones

We found variation in the patterns of divergence at different genomes that reflects their different mutation rates and distribution across populations due to the mode of transmission and inheritance in the species. Mitochondrial genomes (*mt*DNA) of conifers are characterised by an unusually low point mutation rate [108] and restricted gene flow as they are transferred by seeds at relatively short geographical distances (effective migration is no more than 50 m [109]). The *mt*DNA haplotypes were shared between species and their distribution followed geographical rather than species boundaries. This was evident in spatial composition of haplotypes across the species range and further corroborated by the PCA analysis based on *mt*DNA haplotype frequencies, where allopatric populations of all the parental species were significantly overlapping. Additionally, the shared haplotypes were not biased in regard to allopatric or sympatric distribution of the species across their wide ranges. Therefore, the patterns of *mt*DNA polymorphisms suggest incomplete lineage sorting [110, 111] due to relatively recent speciation [50] rather than introgression, as nuclear markers transmitted by pollen over much larger geographical areas showed clear patterns of divergence between species. Such mitotype sharing (both within current contact zones and outside of them), with clear divergence at *cp*DNA markers, was also found in two pine species from southeast China [112]. Incomplete lineage sorting may be common in pines, as those species have long generation time and enormous effective population sizes, both of which significantly lengthen the time required for sorting the ancestral polymorphism [113, 114]. However, discerning the signatures of incomplete lineage sorting from mechanisms such as ancient introgression is often challenging, particularly when these processes act concurrently. Due to the spatial isolation, and highly limited gene flow from allopatric stands, especially in the case of *mt*DNA dispersed with seeds each our hybrid zone is effectively an island and genetic variation of the populations is heavily affected by genetic drift. The isolation was clearly reflected in comparison of *mt*DNA variation between allopatric stands and hybrid zones, where for all species investigated, the variation within hybrid zones was always significantly lower. This effect could be associated with the nonrecombinant, clonal inheritance of mitochondria, resulting in smaller effective population sizes of those genomes. Another consequence of genetic drift is accelerated loss of rare haplotypes from the population, resulting in a reduced number of haplotypes and lower haplotype richness (R_h_). Indeed, the haplotype composition within the hybrid zones, quantified as haplotype richness was substantially lower than in neighbouring reference populations, and this was mainly driven by high frequency of one haplotype (H1 – the most common haplotype in Central European part of Scots pine distribution). Only pines in the Torfowisko pod Zieleńcem contact zone maintained two equally frequent haplotypes across all species, and additionally, this contact zone was characterized by the highest number of haplotypes and haplotype richness among all studied hybrid zones. This was the largest hybrid zone included in this work that maintains the highest *mt*DNA variation. Due to their uniparental mode of transmission, haploid nature, and clonal inheritance, organellar markers have effective population sizes that are four times smaller than those of nuclear markers. This makes them more susceptible to genetic drift, significantly increasing the likelihood of losing rare variants. Such effect was also observed in the case of maternally transmitted chloroplast genome, e.g. in genus *Betula* [31] and explained as the result of asymmetrical hybridization. The effects of genetic drift on *mt*DNA variation could also be detected outside the hybrid zones, but it was only restricted to marginal and presumably preglacial relic populations in Scots pines (3 populations from Tatra and Pieniny Mts.) and to Karkonosze Mts. region in case of *P. mugo*. This region was suggested as potential glacial refugium for other plant species [115] and low nuclear genetic diversity of mountain dwarf pine was previously described there [116]. Notably, our results provide additional support for this hypothesis, as unique, private haplotype (H3) was detected in this area and was reaching the highest frequencies.

The highest number of haplotypes found in the TZ population could alternatively be attributed to recombination during mitochondrial gene flow as discussed in [58] and reported in *Pinus* [117] and other conifers [118–120]. However, our results obtained from the other hybrid zones provide no support for the process of *mt*DNA recombination, as we did not see an excess of singleton haplotypes or novel variants. Either it is a rare event in the hybrid zones or our markers’ resolution is not large enough to detect it.

### Localized effects of hybridization

In our study, similarity between species at *mt*DNA haplotypes was accompanied by significant divergence between species found at the nuclear markers as indicated by clear species division in PCA and individual ancestry coefficients analysis. The pattern is in line with known cytonuclear discordance considered in divergence and evolutionary history [121–127]. No signs of admixture were found in any allopatric populations of the parent species and clear admixture in contact zones suggest that hybridization has localized effect on the genetic variation of the pine species analyzed. Although hybridization acts rather locally, it is worth noting that its effects in plants are not always spatially restricted; in some cases, extensive hybridization can lead to the formation of syngameons, where multiple species form a large hybrid swarm with extensive gene flow [128]. Such scenarios have been observed in various tree species, e.g. oaks, demonstrating that hybridization can significantly impact species boundaries and lead to high levels of genetic diversity and adaptation [129].

We found no signature of reduced genetic diversity at nuclear markers in hybrid zones and distinct pattern of effective population size fluctuations as compared to allopatric stands. The populations of contact zones maintained a similar or slightly higher level of genetic diversity as compared to the reference stands of parental species, but not vastly exceeding it. Initially, an increase of genetic diversity would be expected as a consequence of interspecific gene flow, when recombination leads to formation of new variants resulting from mixing separate gene pools in the hybrid individuals [130, 131]. In the course of time, the combined effect of genetic drift and selection may affect the overall levels of diversity [132] as some new alleles will be eradicated.

Considering the strong effect of genetic drift on *mt*DNA variation and a moderate level of diversity at nuclear markers of the studied contact zones, the results suggest that these sympatric populations are relatively old. The haplotype composition in all species classes of each hybrid zone is unified, exhibiting only slight differences. Additionally, the variance at the nuclear markers is not elevated, which would typically be observed in young sympatric populations founded by two or three separate gene pools. Although the existence of hybrid zones sometimes leads to fear of losing species identity, especially when endangered species cross with more abundant ones [133] or in the cases of anthropogenic impact [134], our case is different. We postulate that the described sympatric populations are of natural origin, as naturally formed hybrid zones might be maintained over longer periods than those established as a result of the anthropogenic disturbance [135]. They also do not threaten allopatric populations as the interspecific gene flow is locally restricted.

### Evolutionary trajectories of the species contact zones

The results indicate mixed ancestry of the hybrids composed of different contributions of parental species and suggests that introgression may play an important role in adaptation of hybrids to specific environments in the contact zones. Individual ancestry coefficients analysis defines two separate genetic clusters in every contact zone, associated with parental taxa ancestry (*mugo* vs. *sylvestris*), even in the populations with *P. uliginosa* individuals present (BS, TZ), though proportions of ancestry vary between different contact zones. Our data does not separate the peat-bog pine from mountain dwarf pine clearly, what results from their close genetic relations and reticulate events in the divergence of *P. uliginosa* [51]. Each of the selected hybrid zones have different history, but all of them seems to be facing the same destiny – loss of the genetic diversity and slow extinction caused mainly by drought. However, the population from Torfowisko pod Zieleńcem grows in the similar environment and exhibits the highest variety of mitochondrial DNA with the most frequent haplotype H30 characteristic for *locus classicus* of the Polish peat-bog pines – Wielkie Torfowisko Batorowskie reserve [57, 60, 136]. While the phenotype of the trees selected in TZ as peat-bog pines matched perfectly the phenotype of Batorów pines, many of the hybrids from TZ resembled intermediate forms between *P. mugo*, *P. uliginosa* and *P. sylvestris*, what is consistent with previous morphology isoenzyme and molecular analyses [50, 137–141]. These premises indicates that some of the contact zones of pines may be the origin of speciation leading to forming of *Pinus uliginosa*. Results obtained from Bór na Czerwonem (BC) and Czarny Dunajec (CD) leads to the opposite impression, as there are no phenotypic peat-bog pines and the mitochondrial genetic makeup of those populations is unified and the nuclear genome pools are probably influenced by nearby Scots pine forests, which surrounds these contact zones. If the peat-bog pines would be a result of hybrid speciation occurring in the contact zones of Scots pine and mountain dwarf pine, it would probably require isolation from the outside Scots pine pollen, as it is in the case of TZ and Batorów reserve.

As we estimated here the neutral genetic variance, more thorough research involving adaptive markers is crucial to better understand the function of these hybrid zones and evaluate mechanisms of selection at the molecular level. Our research demonstrates that the hybrid zones of pines can be very useful in evolutionary studies of forest trees and therefore protection strategies should be considered [142].

## Conclusions

Our results suggest that hybrid zones of the species are sinks rather than melting pots of genetic diversity in the analysed species, especially in the case of mitochondrial genome. Distribution of *mt*DNA haplotypes follows geographical rather than species boundaries and shows reduced diversity in hybrid zones due to genetic drift. Clear signatures of species divergence were observed at *n*DNA between allopatric stands. Although the hybridization heavily impacts the sympatric populations, it is limited only to the contact zones, as we do not observe admixed individuals in allopatric populations nor mitochondrial haplotypes restricted only to the hybrid zones. The spectrum of parental species ancestry in hybrids suggests old evolutionary history of the investigated sympatric populations. The result indicates that the introgression may play an important role in adaptation of hybrids to specific environments.

## Electronic supplementary material

Below is the link to the electronic supplementary material.

Supplementary Material 1

## Data Availability

The datasets generated and/or analysed during the current study are available in the Dryad repository: DOI: 10.5061/dryad.gf1vhhmxn.
