# Supplementary material for: Hybridization has localized effect on genetic variation in closely related pine species
Source: BMC Plant Biol. 2024 Oct 26;24:1007. doi: 10.1186/s12870-024-05732-y (PMC11520059; doi:10.1186/s12870-024-05732-y)
Supplement: Supplementary file 1 — Supplementary Material 1 [file 12870_2024_5732_MOESM1_ESM.docx]

**Fig. S1.** The distribution ranges of *P. sylvestris* (light blue) and *P. mugo* (yellow) in Europe are shown. Light blue points denote isolated populations of Scots pine. Note that two species are also separeted by elevation (Scots pine: 0 - 1000 m asl., dwarf mountain pine 1,100 - 2,200 m asl.) and this is not included here. The distribution of species were adapted from: Caudullo et.al (2024), “Chorological data for the main European woody species”, Mendeley Data, V18, doi: 10.17632/hr5h2hcgg4.18.


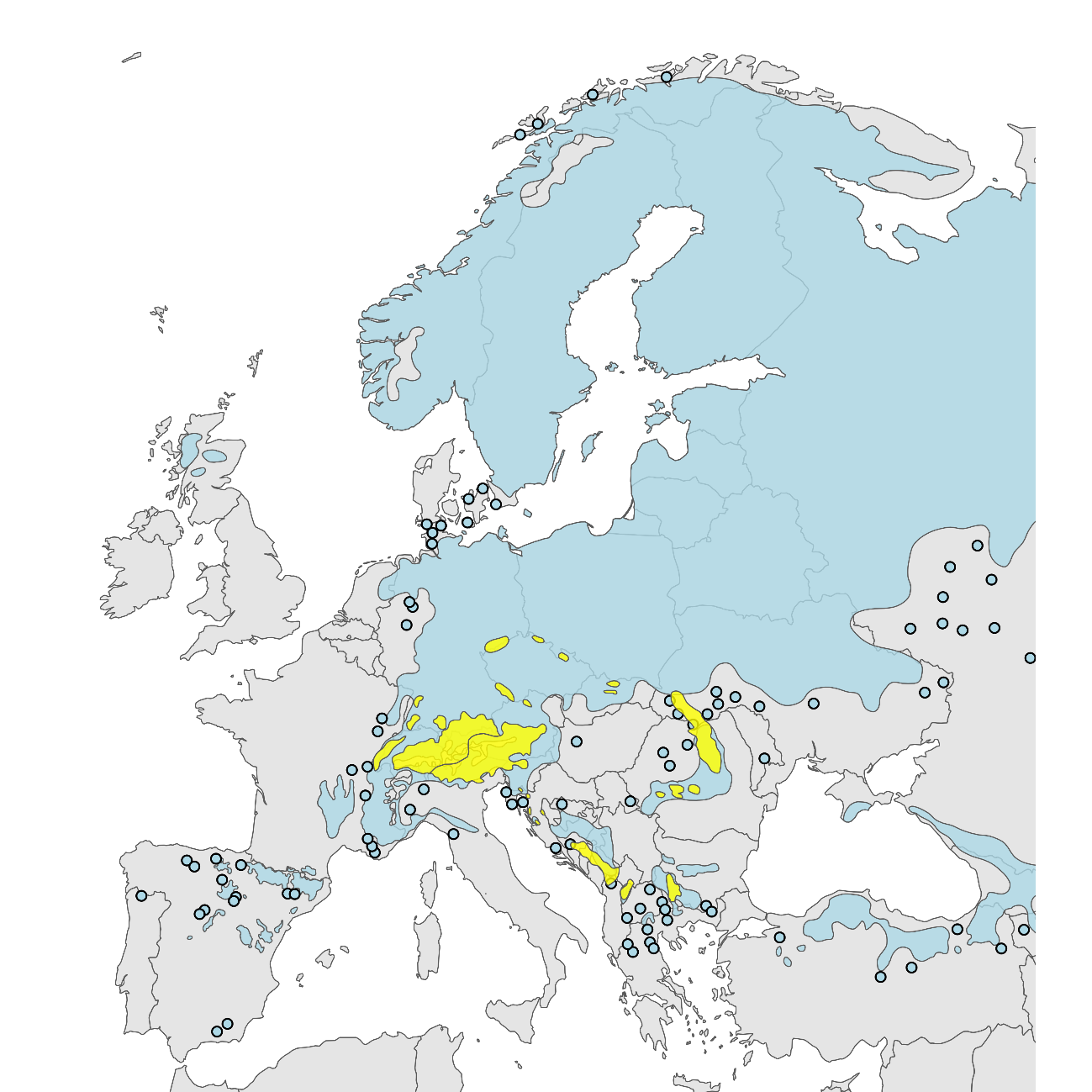


**Fig. S2.** Results of PCA analysis on population level based on mitochondrial DNA haplotypes frequencies. Populations are colored according to species identification (A) emphasizing the hybrid classification; (B) emphasizing the hybrid zone origin


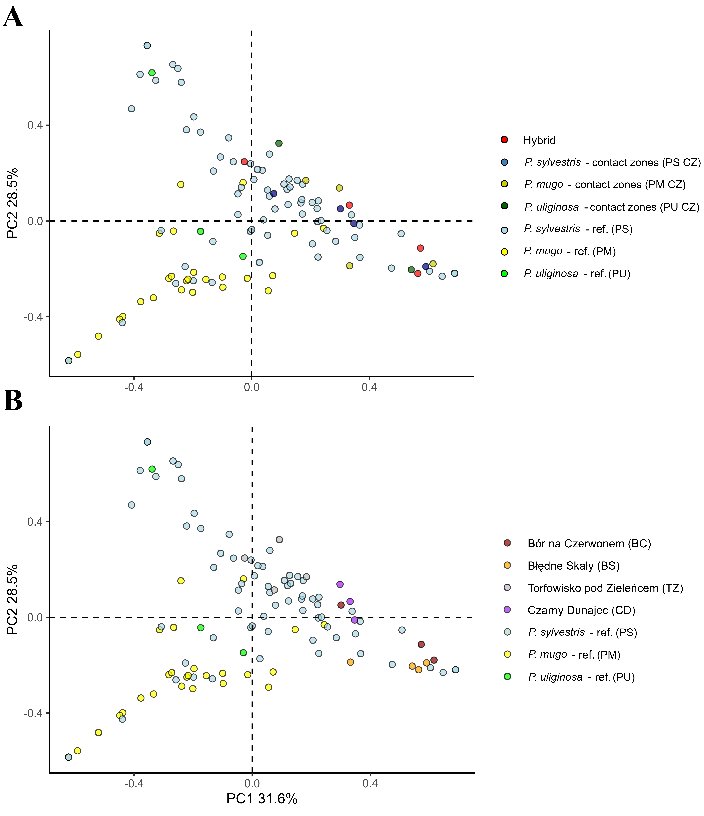


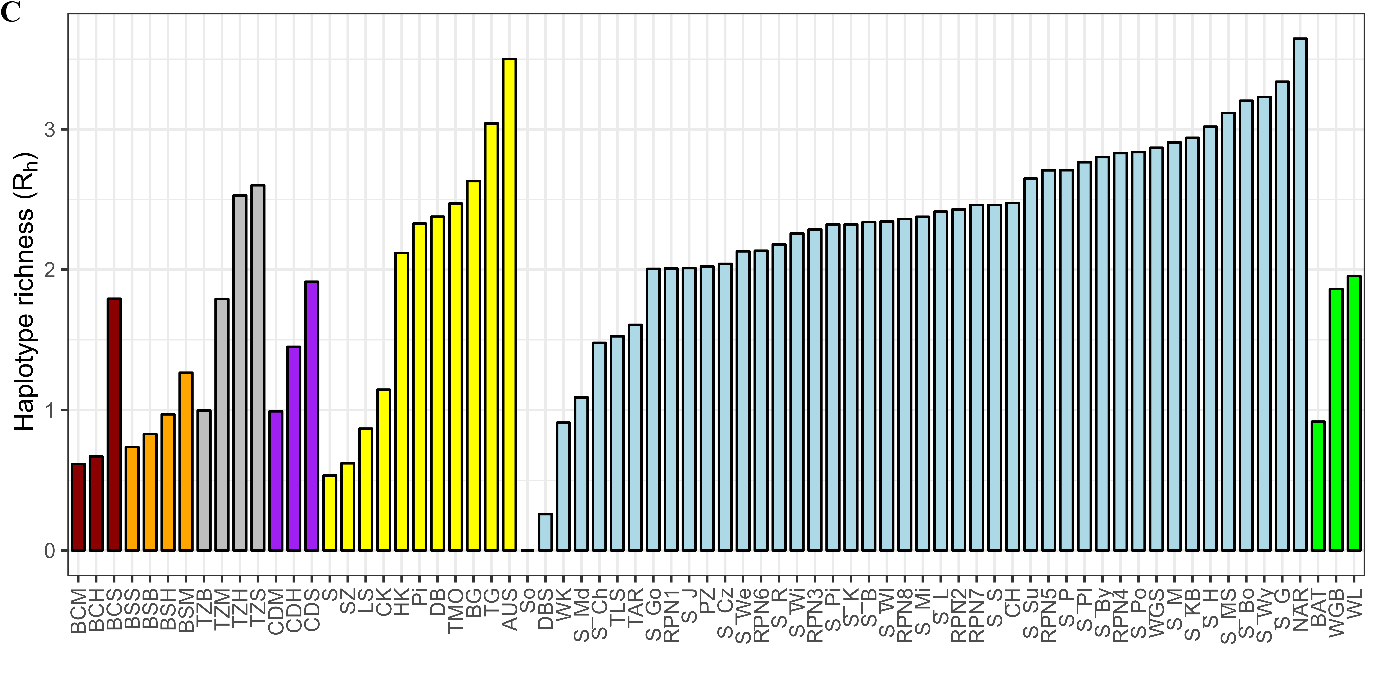

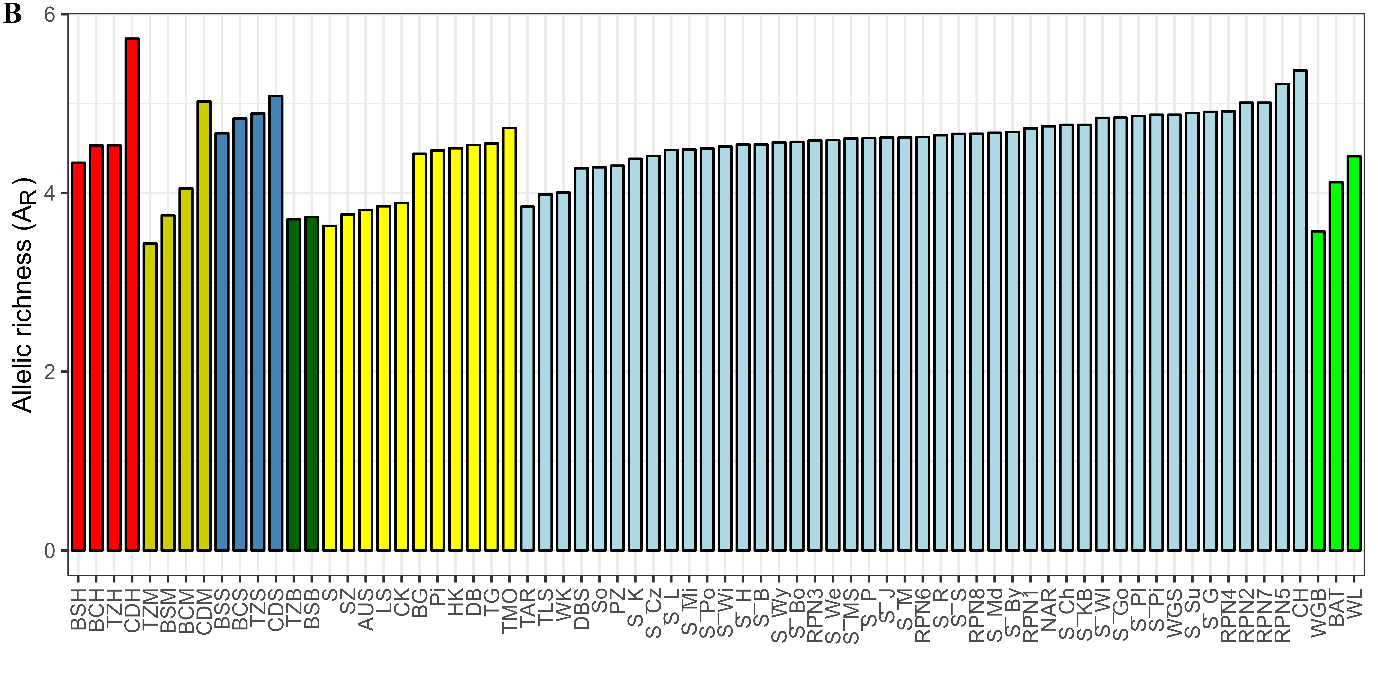

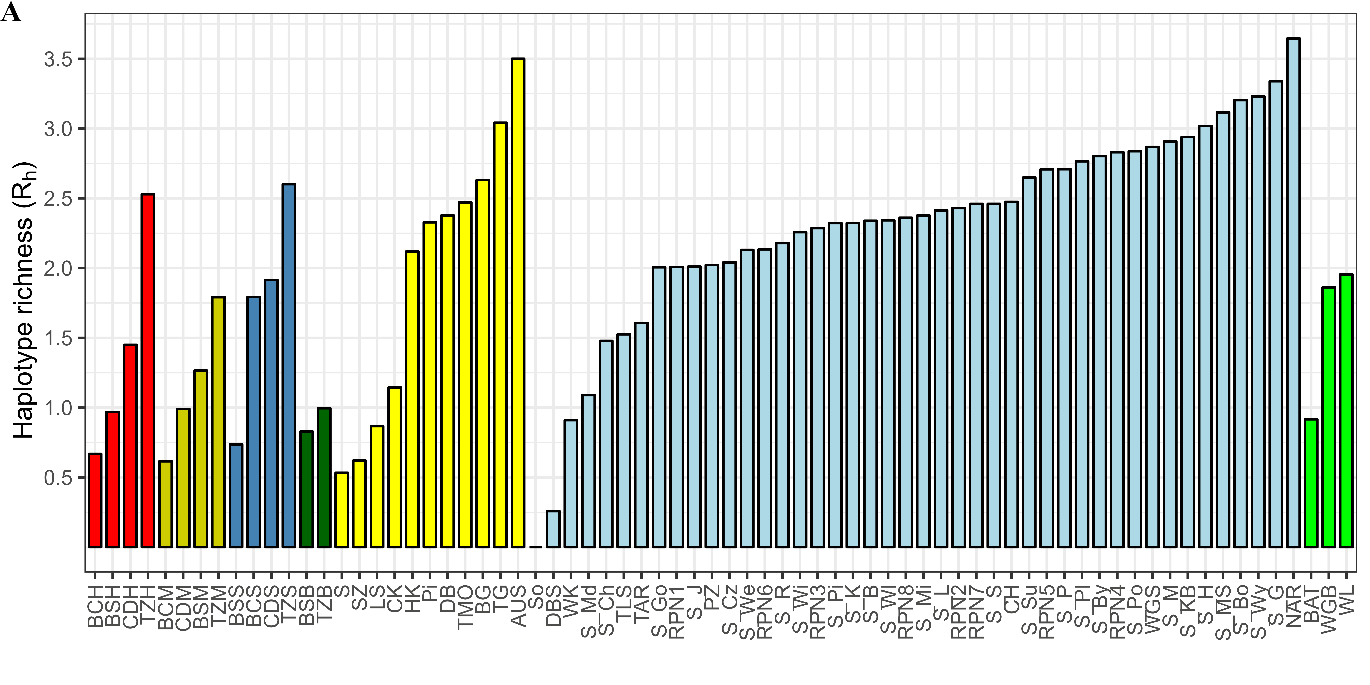
**Fig. S3.** Genetic diversity of selected pine populations. A, C - mitochondrial haplotype richness (R_h_) estimated for studied populations. B, D - nuclear allelic richness (A_R_) estimated for studied populations.


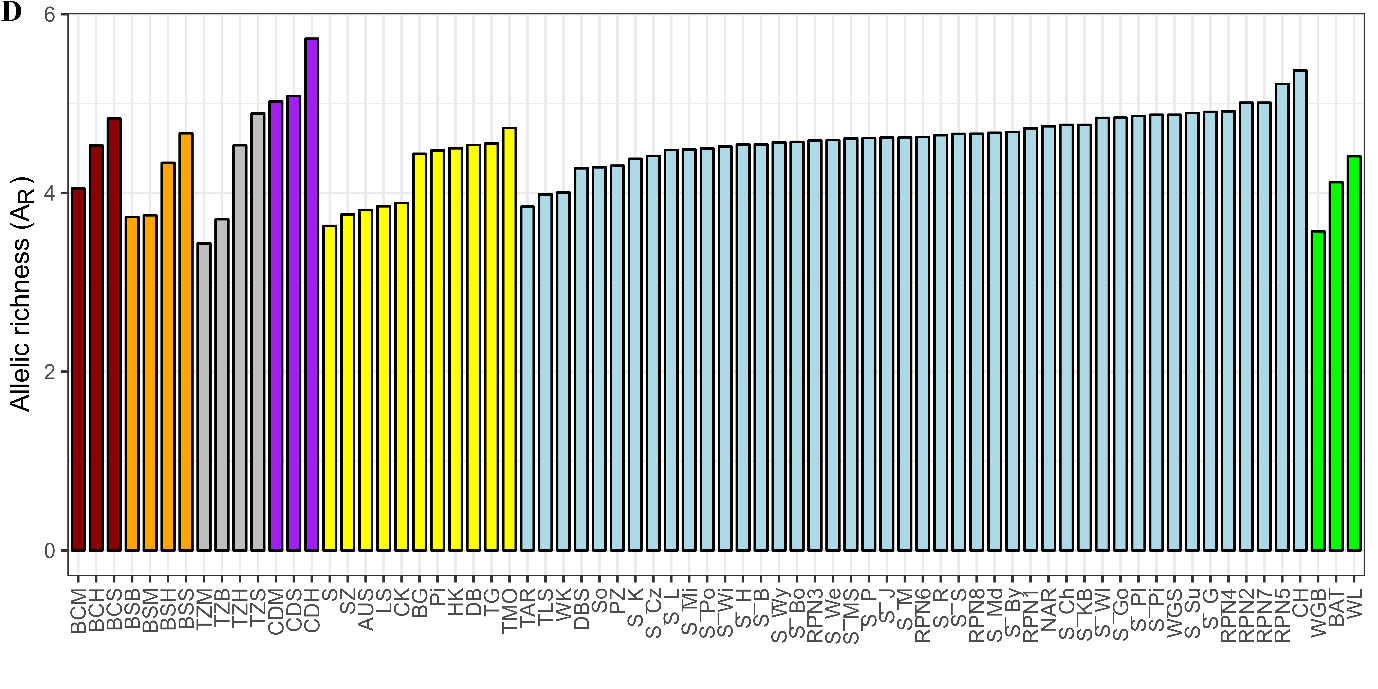


**Fig. S4.** Principal Coordinate Analysis (PCoA) based on Nei genetic distance between all populations, derived from nuclear microsatellite data (*n*SSR).


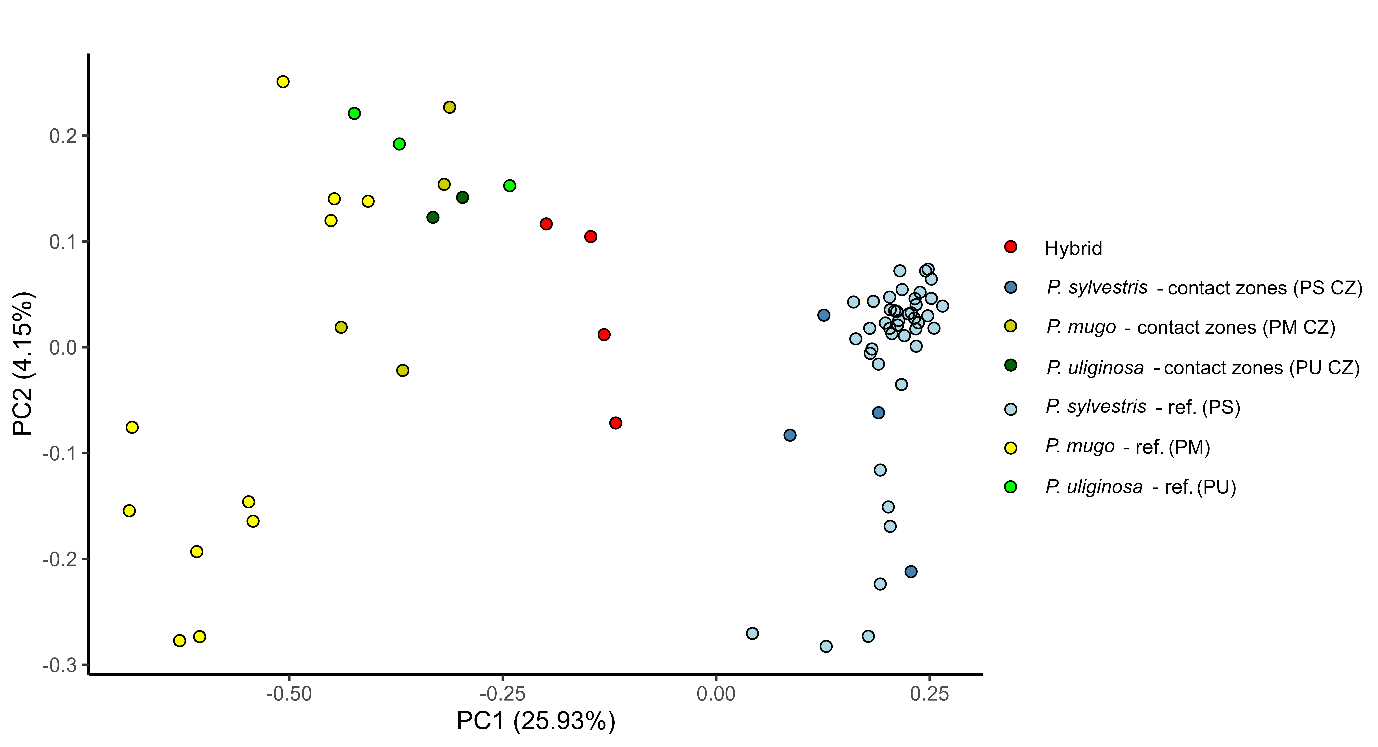


**Fig. S5.** Cross entropy between ten different runs for each K in LEA plotted vs number of ancestral populations. The optimal number of clusters is detected by first significant drop of cross entropy at K = 2.


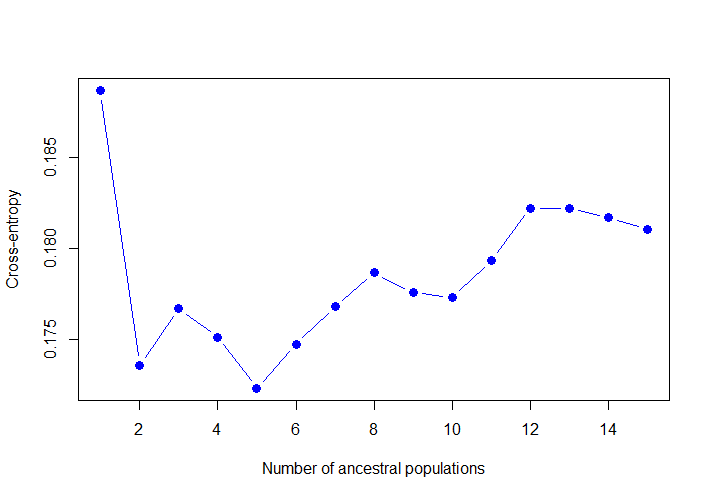


**Fig. S6.** Results of the individual ancestry coefficients analysis for K=2 derived from nuclear microsatellite data (*n*SSR), for all studied populations. Individuals clustered to *P. sylvestris* genetic group are marked in blue, and to *P. mugo* populations in yellow.


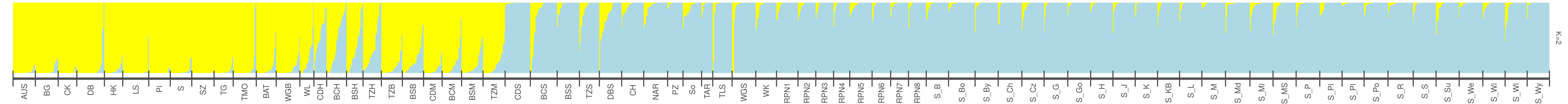


**Fig. S7.** Results of the individual ancestry coefficients analysis for K=3 – K=7 derived from nuclear microsatellite data (*n*SSR), for all studied populations


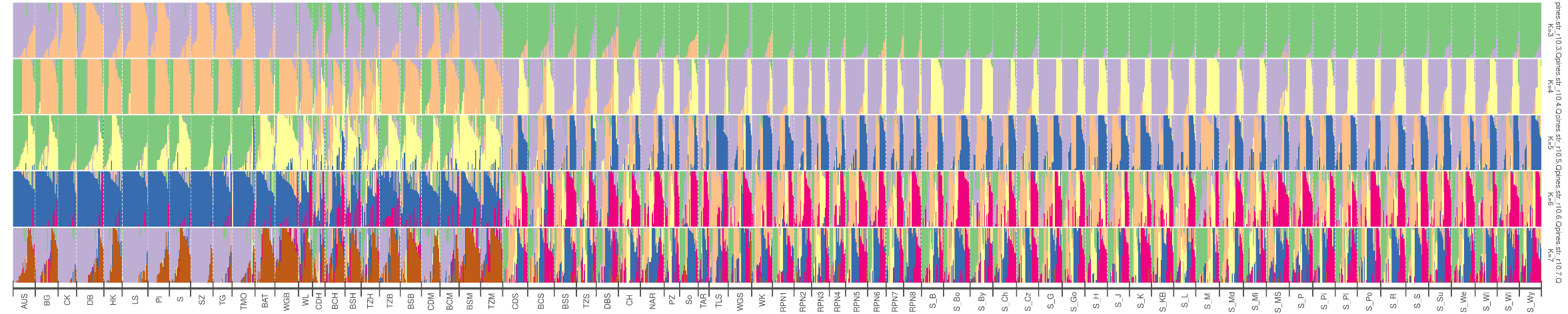


**Table S1.** Localization of four contact zones of studied pines and reference allopatric populations of parental taxa.

|  | **Acronym** | **N** | **Population/group** | **Longitude (E)** | **Latitude (N)** |
| --- | --- | --- | --- | --- | --- |
| Bór na Czerwonem  contact zone | BCH | 27 | Poland, Bór na Czerwonem; hybrids | 20°2'23.64" | 49°27'37.44" |
|  | BCM | 26 | Poland, Bór na Czerwonem; *P. mugo* | 20°2'23.64" | 49°27'37.44" |
|  | BCS | 36 | Poland, Bór na Czerwonem; *P. sylvestris* | 20°2'23.64" | 49°27'37.44" |
| Błędne Skały  contact zone | BSB | 29 | Poland, Błędne Skały; *P. uliginosa* | 16°17'15.72" | 50°28'49.44" |
|  | BSH | 22 | Poland, Błędne Skały; hybrids | 16°17'15.72" | 50°28'49.44" |
|  | BSM | 29 | Poland, Błędne Skały; *P. mugo* | 16°17'15.72" | 50°28'49.44" |
|  | BSS | 30 | Poland, Błędne Skały; *P. sylvestris* | 16°17'15.72" | 50°28'49.44" |
| Czarny Dunajec  contact zone | CDH | 17 | Poland, Czarny Dunajec; hybrids | 19°49'6.6" | 49°28'11.28" |
|  | CDM | 24 | Poland, Czarny Dunajec; *P. mugo* | 19°49'6.6" | 49°28'11.28" |
|  | CDS | 34 | Poland, Czarny Dunajec; *P. sylvestris* | 19°49'6.6" | 49°28'11.28" |
| Torfowisko pod Zieleńcem  contact zone | TZB | 28 | Poland, Torfowisko pod Zieleńcem; *P. uliginosa* | 16°24'45" | 50°20'50.28" |
|  | TZH | 25 | Poland, Torfowisko pod Zieleńcem; hybrids | 16°24'45" | 50°20'50.28" |
|  | TZM | 30 | Poland, Torfowisko pod Zieleńcem; *P. mugo* | 16°24'45" | 50°20'50.28" |
|  | TZS | 27 | Poland, Torfowisko pod Zieleńcem; *P. sylvestris* | 16°24'45" | 50°20'50.28" |
| Allopatric reference *P. mugo* | AUS | 30 | Austria, Berchtesgaden National Park | 15°1'23.16" | 47°57'28.8" |
|  | BG | 31 | Poland, Babia Góra | 19°31'50.88" | 49°34'23.52" |
|  | CK | 26 | Poland, Karkonosze Mountains, Czarny Kocioł | 15°35'5.64" | 50°47'18.6" |
|  | DB | 36 | Poland, Tatra Mountains, Dolina Białego | 19°57'12.24" | 49°16'1.2" |
|  | HK | 31 | Poland, Tatra Mountains, Hala Kondratowa | 19°56'48.48" | 49°14'51" |
|  | LS | 35 | Poland, Karkonosze Mountains, Łabski Szczyt | 15°31'53.04" | 50°47'26.52" |
|  | PI | 30 | Poland, Pilsko | 19°19'1.56" | 49°31'44.4" |
|  | S | 30 | Poland, Karkonosze Mountains, Śnieżka | 15°44'24.72" | 50°44'11.76" |
|  | SZ | 30 | Poland, Karkonosze Mountains, Szrenica | 15°30'54.36" | 50°47'33" |
|  | TG | 30 | Poland, Tatra Mountains, Grześ | 19°45'59.76" | 49°14'12.12" |
|  | TMO | 30 | Poland, Tatra Mountains, Morskie Oko | 20°4'4.8" | 49°12'0.72" |
|  | M1^*^ | 12 | Poland, Sudetes, Slaskie Kamienie & Czarny Kociol Jagniatkowski | 15°36'08" | 50°46'35" |
|  | M2^*^ | 12 | Poland, Tatra Mountains, Piec Stawow Polskich | 20°03'05" | 49°13'09" |
|  | M3^*^ | 12 | Slovakia, Lower Tatra Mts., Lysa above Jasna | 19°39'35" | 49°00'43" |
|  | M4^*^ | 12 | Ukraine, Gorgany Mountains, Osmoloda | 23°55'19" | 48°40'33" |
|  | M5^*^ | 12 | Romania, Muntii Rodnei, Pasul Prislop | 24°48'00" | 47°34'03" |
|  | M6^*^ | 11 | Romania, Muntii Bucegi, Busteni | 25°27'06" | 45°25'55" |
|  | M7^*^ | 12 | Bulgaria, Rila Mountains, Belica | 23°30'00" | 42°04'01" |
|  | M8^*^ | 9 | Bulgaria, Pirin Mountains, Vikhren | 23°25'22" | 41°46'07" |
|  | M9^*^ | 12 | Montenegro, Durmitor Mountains, Zhabjak | 19°05'27" | 43°09'33" |
|  | M10^*^ | 12 | Bosnia and Herzegovina, Bjelasnica Mountain near Sarajevo | 18°13'08" | 43°45'00" |
|  | M11^*^ | 11 | Slovenia, Kamnik–Savinja Alps, Kamniska Bistrica | 14°37'51" | 46°05'10" |
|  | M12^*^ | 12 | Austria/Italy, Karnishe Alps, Passo di Pramollo | 13°15'35" | 46°32'45" |
|  | M13^*^ | 12 | Germany, Bavarian Alps, Kreuzspitze | 10°55'12" | 47°31'30" |
|  | M14^*^ | 12 | Austria, Karwendel Gebirge, Scharnitz | 11°17'45" | 47°22'42" |
|  | M15^*^ | 12 | Italy, Dolomites, Brenta Group, Lago di Tovel | 10°56'46" | 46°15'39" |
|  | M16^*^ | 12 | Italy/France, Maritime Alps, Coll de Tende | 07°22'30" | 44°08'00" |
| Allopatric reference *P. sylvestris* | CH | 30 | Poland, Chojnik | 15°38'26.52" | 50°50'18.96" |
|  | DBS | 31 | Poland, Tatra Mountains, Dolina Białego | 19°57'29.88" | 49°15'53.28" |
|  | NAR | 32 | Poland, Stołowe Mountains, Narożnik | 16°20'51.72" | 50°27'31.68" |
|  | PZ | 29 | Poland, Polanica-Zdrój | 16°29'51.72" | 50°23'52.08" |
|  | RPN1 | 29 | Poland, Roztocze National Park, Bukowa Góra | 22°58'8.04" | 50°36'4.32" |
|  | RPN2 | 24 | Poland, Roztocze National Park, Czarny Staw | 22°59'0.6" | 50°35'9.24" |
|  | RPN3 | 24 | Poland, Roztocze National Park, Kruglik | 23°1'29.28" | 50°33'8.28" |
|  | RPN4 | 22 | Poland, Roztocze National Park, Panasówka | 22°55'17.76" | 50°36'3.6" |
|  | RPN5 | 30 | Poland, Roztocze National Park, Słupy | 23°1'49.08" | 50°36'4.68" |
|  | RPN6 | 25 | Poland, Roztocze National Park, Słupy | 23°2'44.52" | 50°36'16.92" |
|  | RPN7 | 24 | Poland, Roztocze National Park, Jarugi | 23°3'43.92" | 50°38'17.88" |
|  | RPN8 | 24 | Poland, Roztocze National Park, Czarny Wygon | 23°3'39.96" | 50°39'46.08" |
|  | SO | 30 | Poland, Pieniny Mountains, Sokolica | 20°26'29.04" | 49°25'6.24" |
|  | TAR | 20 | Poland, Tarnawa | 22°49'46.92" | 49°6'45.72" |
|  | TLS | 30 | Poland, Tatra Mountains, Łysa Skałka | 20°6'48.96" | 49°15'53.64" |
|  | WGS | 32 | Poland, Węgliniec | 15°13'35.04" | 51°17'39.12" |
|  | WK | 28 | Poland, Tatra Mountains, Koryciska Wielkie | 19°48'30.24" | 49°16'10.56" |
|  | S_B | 30 | Poland, Barlinek | 15°13'58.8" | 52°59'52.44" |
|  | S_G | 30 | Poland, Gubin | 14°41'9.96" | 51°56'30.84" |
|  | S_GO | 30 | Poland, Goleniów | 14°49'45.12" | 53°33'49.68" |
|  | S_R | 30 | Poland, Rychtal | 17°50'49.2" | 51°8'31.92" |
|  | S_BY | 31 | Poland, Bytów | 17°29'55.68" | 54°10'39" |
|  | S_CZ | 30 | Poland, Czarne | 16°56'38.4" | 53°42'13.32" |
|  | S_K | 30 | Poland, Kaliska | 18°13'6.24" | 53°54'20.88" |
| Allopatric reference *P. sylvestris* | S_BO | 30 | Poland, Bolewice | 16°7'4.08" | 52°23'50.28" |
|  | S_M | 29 | Poland, Milicz | 17°16'0.48" | 51°31'2.64" |
|  | S_S | 30 | Poland, Spała | 20°8'30.12" | 51°32'28.68" |
|  | S_KB | 30 | Poland, Kobiór | 18°56'2.4" | 50°3'43.92" |
|  | S_MD | 30 | Poland, Międzylesie | 16°39'17.64" | 50°8'51.72" |
|  | S_P | 30 | Poland, Prószków | 17°52'13.08" | 50°34'21.72" |
|  | S_CH | 30 | Poland, Chełmiec | 20°39'49.32" | 49°37'55.56" |
|  | S_J | 29 | Poland, Janów Lubelski | 22°24'42.12" | 50°42'0" |
|  | S_L | 30 | Poland, Lipnica | 17°24'26.64" | 53°59'49.92" |
|  | S_H | 29 | Poland, Hajnówka | 23°34'38.64" | 52°44'15.36" |
|  | S_MS | 29 | Poland, Międzyrzec | 22°46'27.48" | 51°58'50.88" |
|  | S_PI | 29 | Poland, Pisz-Dziadki | 21°37'21.36" | 53°30'11.52" |
|  | S_SU | 30 | Poland, Supraśl | 23°20'6.72" | 53°12'26.64" |
|  | S_PL | 30 | Poland, Płońsk | 20°22'1.92" | 52°37'36.12" |
|  | S_WE | 30 | Poland, Węgliniec | 15°13'44.76" | 51°18'1.44" |
|  | S_WI | 30 | Poland, Wichrowo | 20°25'49.08" | 54°1'34.68" |
|  | S_WY | 30 | Poland, Wyszków | 21°26'29.04" | 52°35'45.96" |
|  | S_MI | 30 | Poland, Miłomłyn | 19°49'37.56" | 53°46'3.72" |
|  | S_PO | 30 | Poland, Pomorze | 20°41'57.12" | 52°52'22.44" |
|  | S_WL | 28 | Poland, Włocławek | 19°3'4.32" | 52°39'9.36" |
|  | S_EST^*^ | 10 | Estonia, Vardi | 25°32'35.52" | 58°18'56.16" |
|  | S_S4^*^ | 15 | Serbia, Velika Plec | 20°0'48.24" | 44°6'0.36" |
|  | S_LT2^*^ | 15 | Lithuania, Ciapkeliai | 24°31'2.28" | 54°0'2.88" |
|  | S_CH1^*^ | 10 | Switzerland, Follateres | 7°5'8.16" | 46°8'5.28" |
|  | S_G3^*^ | 8 | Greece, Pteri, Ritni | 22°4'21.36" | 38°8'58.2" |
|  | S_HU^*^ | 15 | Hungary, Supron | 16°31'12" | 47°40'12" |
|  | S_F21^*^ | 9 | Finland, Sella Reindeer Park Sallon Poropuisto | 28°49'27.84" | 66°44'20.04" |
|  | S_F24^*^ | 10 | Finland -Kajaanintie 182 Kuusamo | 29°14'13.2" | 65°48'29.16" |
|  | S_F12^*^ | 9 | Finland, Kaamasentie 2330 Inarii | 27°12'44.28" | 69°10'48" |
|  | S_F1^*^ | 12 | Finland, Joutsa | 26°8'24" | 61°44'24" |
|  | S_F2^*^ | 11 | Finland, Temmes and Tyrnava | 25°42'36" | 64°41'24" |
|  | S_F3^*^ | 12 | Finland, Rovaniemi | 26°12'36" | 66°34'12" |
|  | S_F4^*^ | 10 | Finland, Kielajoki | 29°4'12" | 69°39'0" |
|  | S_U2^*^ | 9 | Ukraine, Yavorivsky National Park | 23°32'48.48" | 50°0'56.16" |
|  | S_R12^*^ | 10 | Russia- Volga Kama Nature Reserve | 49°17'21.12" | 55°18'9.36" |
|  | S_R1^*^ | 10 | Russia, Bobrovka | 83°52'48" | 53°10'12" |
|  | S_FR1^*^ | 10 | France, Arlac Mayers | 3°41'24" | 45°24'0" |
|  | S_D1^*^ | 10 | Germany, Godendorf | 13°7'48" | 53°16'12" |
|  | S_N2^*^ | 15 | Norway, Ostmarka | 11°3'0" | 59°52'12" |
|  | S_N3^*^ | 15 | Norway, Smoldalen | 12°25'12" | 61°27'0" |
|  | S_LVA^*^ | 9 | Latvia, Dunezers Lake | 24°21'0" | 57°9'0" |
|  | S_RUS7^*^ | 10 | Russia, Yakkima | 30°8'24" | 61°31'12" |
| Allopatric reference *P. uliginosa* | BAT | 15 | Poland, Wielkie Torfowisko Batorowskie | 16°23'1.68" | 50°27'29.16" |
|  | WGB | 32 | Poland, Węgliniec | 15°13'35.04" | 51°17'39.12" |
|  | WL | 19 | Poland, Węglowiec | 15°11'29.4" | 51°17'52.8" |

N – number of individuals sampled, ^*^ - reference population used only for *mt*DNA analysis, see main text for details

**Table S2.** Haplotypes H1-H30 with corresponding sequence comprised of 13 *mt*DNA markers divided in two multiplexes.

|  |  | **Multiplex I** | | | | | | | **Multiplex II** | | | | | |
| --- | --- | --- | --- | --- | --- | --- | --- | --- | --- | --- | --- | --- | --- | --- |
| Haplotype | N | PR5 | PR7 | PR1 | PR19 | PR20 | PR21 | PR24 | PR25 | PR29 | PR30 | PR31 | PR32 | nad1 |
| H1 | 973 | G | C | G | T | C | G | T | G | G | G | G | G | G |
| H30 | 677 | T | A | T | G | A | T | T | T | T | G | T | G | G |
| H11 | 246 | G | A | G | T | C | G | T | G | G | G | G | G | G |
| H15 | 215 | G | A | G | G | C | G | T | G | G | G | G | G | G |
| H3 | 117 | G | C | G | T | C | G | T | G | G | G | G | T | G |
| H6 | 99 | G | C | G | G | C | G | T | G | G | G | G | G | G |
| H22 | 45 | G | A | T | G | A | T | T | T | T | G | T | G | G |
| H28 | 40 | T | A | T | G | A | G | T | T | T | G | T | G | G |
| H21 | 35 | G | A | T | G | A | G | T | T | T | G | T | G | G |
| H26 | 28 | T | A | T | T | A | T | T | T | T | G | T | G | G |
| H18 | 22 | G | A | G | G | C | G | T | T | G | G | G | G | G |
| H9 | 18 | G | C | G | G | C | G | G | G | G | G | G | G | G |
| H13 | 14 | G | A | G | T | C | G | T | T | G | G | G | G | G |
| H25 | 14 | T | A | G | G | C | G | T | G | G | G | G | G | G |
| H8 | 8 | G | C | G | G | C | G | T | G | G | T | G | G | G |
| H17 | 6 | G | A | G | G | C | G | T | G | G | T | G | G | G |
| H23 | 6 | T | C | G | T | C | G | T | G | G | G | G | G | G |
| H5 | 5 | G | C | G | T | C | T | T | G | G | G | G | G | G |
| H10 | 5 | G | C | G | G | C | G | G | G | G | T | G | G | G |
| H12 | 5 | G | A | G | T | C | G | T | G | G | T | G | G | G |
| H19 | 5 | G | A | G | G | C | G | T | T | G | T | G | G | G |
| H2 | 3 | G | C | G | T | C | G | T | G | G | G | G | G | C |
| H24 | 3 | T | A | G | T | C | G | T | G | G | G | G | G | G |
| H29 | 3 | T | A | T | G | A | T | T | G | G | G | G | G | G |
| H4 | 2 | G | C | G | T | C | G | G | G | G | G | G | G | G |
| H31 | 2 | T | A | T | G | A | T | T | T | T | G | T | G | C |
| H7 | 1 | G | C | G | G | C | G | T | G | G | G | G | G | C |
| H14 | 1 | G | A | G | T | C | T | T | G | G | G | G | G | G |
| H16 | 1 | G | A | G | G | C | G | T | G | G | G | G | G | C |
| H20 | 1 | G | A | T | G | A | G | T | G | G | G | G | G | G |
| H27 | 1 | T | A | T | G | A | G | T | T | T | G | G | G | G |

N – number of individuals with certain haplotype

**Table S3.** Mean values of basic genetic statistics for different groups of populations.

|  | ***n*SSR** | | | | | | ***mt*DNA** | | | | |
| --- | --- | --- | --- | --- | --- | --- | --- | --- | --- | --- | --- |
| **group** | **N** | **A** | **A_R_** | **H_o_** | **H_e_** | **F** | **N** | **H_A_** | **N_e_** | **R_h_** | **H_e_** |
| Hybrid | 22.750 | 73.250 | 4.783 | 0.487 | 0.563 | 0.135 | 22.750 | 3.250 | 1.888 | 1.405 | 0.420 |
| *P. sylvestris* – CZ. | 33.000 | 84.500 | 4.865 | 0.473 | 0.534 | 0.121 | 33.000 | 4.500 | 2.395 | 1.828 | 0.518 |
| *P. mugo* – CZ. | 27.500 | 64.750 | 4.064 | 0.430 | 0.497 | 0.144 | 27.250 | 3.250 | 1.879 | 1.166 | 0.444 |
| *P. uliginosa* – CZ. | 28.500 | 58.000 | 3.718 | 0.419 | 0.477 | 0.121 | 28.500 | 2.000 | 1.680 | 0.912 | 0.402 |
| *P. sylvestris* – ref. | 29.136 | 78.047 | 4.631 | 0.483 | 0.511 | 0.052 | 29.674 | 5.372 | 2.796 | 2.283 | 0.623 |
| *P. mugo* – ref. | 29.818 | 68.909 | 4.197 | 0.409 | 0.489 | 0.150 | 30.818 | 4.545 | 2.597 | 1.967 | 0.529 |
| *P. uliginosa* – ref. | 26.000 | 63.667 | 4.034 | 0.439 | 0.491 | 0.107 | 29.667 | 4.000 | 1.908 | 1.578 | 0.456 |
| Bór na Czerwonem | 29.667 | 74.667 | 4.471 | 0.460 | 0.528 | 0.141 | 29.667 | 3.333 | 1.537 | 1.026 | 0.305 |
| Błędne Skały | 27.500 | 64.500 | 4.121 | 0.450 | 0.507 | 0.138 | 27.500 | 2.750 | 1.499 | 0.950 | 0.318 |
| Torfowisko pod Zieleńcem | 27.500 | 67.000 | 4.140 | 0.471 | 0.500 | 0.047 | 27.500 | 4.500 | 2.639 | 1.979 | 0.630 |
| Czarny Dunajec | 25.333 | 84.667 | 5.278 | 0.458 | 0.580 | 0.212 | 25.000 | 3.333 | 1.991 | 1.452 | 0.519 |

N – number of samples; A – number of alleles; A_R_ – allelic richness; H_o_ – observed heterozygosity; H_e_ – expected heterozygosity; F – fixation index; H_A_ – number of haplotypes; N_e_ – effective number of haplotypes; R_h_ – haplotype richness; H_e_ – haplotype variation.

**Table S4.** Basic genetic statistics scored for nuclear (*n*SSR) and mitochondrial (*mt*DNA) markers.

|  |  | ***n*SSR** | | | | | | | | ***mt*DNA** | | | | |
| --- | --- | --- | --- | --- | --- | --- | --- | --- | --- | --- | --- | --- | --- | --- |
|  | **pop** | **N** | **A** | **A_R_** | **H_o_** | **H_e_** | **F** | **N_E_** | **B. p.v.** | **N** | **H_A_** | **N_e_** | **R_h_** | **H_e_** |
| Bór na Czerwonem  contact zone | BCH | 27 | 72 | 4.533 | 0.478 | 0.533 | 0.121 | 196.1 | 0.286 | 27 | 2 | 1.246 | 0.669 | 0.205 |
|  | BCM | 26 | 66 | 4.049 | 0.39 | 0.491 | 0.206 | 2000 | 0.108 | 26 | 3 | 1.17 | 0.615 | 0.151 |
|  | BCS | 36 | 86 | 4.831 | 0.512 | 0.559 | 0.097 | 412.6 | 0.352 | 36 | 5 | 2.197 | 1.793 | 0.56 |
| Błędne Skały  contact zone | BSB | 29 | 57 | 3.73 | 0.418 | 0.496 | 0.173 | 51.5 | 0.36 | 29 | 2 | 1.399 | 0.829 | 0.296 |
|  | BSH | 22 | 66 | 4.337 | 0.475 | 0.539 | 0.119 | 113.9 | 0.4 | 22 | 3 | 1.322 | 0.97 | 0.255 |
|  | BSM | 29 | 58 | 3.75 | 0.455 | 0.486 | 0.142 | 2000 | 0.316 | 29 | 3 | 2.046 | 1.266 | 0.53 |
|  | BSS | 30 | 77 | 4.668 | 0.453 | 0.505 | 0.116 | 203.8 | **0.026** | 30 | 3 | 1.226 | 0.736 | 0.191 |
| Czarny Dunajec  contact zone | CDH | 17 | 83 | 5.726 | 0.487 | 0.643 | 0.26 | 2000 | 0.327 | 17 | 3 | 1.966 | 1.45 | 0.522 |
|  | CDM | 25 | 81 | 5.023 | 0.421 | 0.545 | 0.216 | 2000 | **0.009** | 24 | 2 | 1.882 | 0.991 | 0.489 |
|  | CDS | 34 | 90 | 5.085 | 0.465 | 0.553 | 0.16 | 2000 | **0.038** | 34 | 5 | 2.125 | 1.914 | 0.545 |
| Torfowisko pod Zieleńcem  contact zone | TZB | 28 | 59 | 3.706 | 0.42 | 0.458 | 0.068 | 189.3 | 0.216 | 28 | 2 | 1.96 | 0.996 | 0.508 |
|  | TZH | 25 | 72 | 4.534 | 0.508 | 0.535 | 0.041 | 330.1 | 0.345 | 25 | 5 | 3.019 | 2.529 | 0.697 |
|  | TZM | 30 | 54 | 3.433 | 0.455 | 0.467 | 0.01 | 150.4 | 0.254 | 30 | 5 | 2.419 | 1.791 | 0.607 |
|  | TZS | 27 | 83 | 4.888 | 0.502 | 0.539 | 0.069 | 2000 | 0.086 | 27 | 6 | 3.156 | 2.601 | 0.709 |
| Allopatric reference *P. mugo* | AUS | 30 | 58 | 3.808 | 0.442 | 0.463 | 0.007 | 2000 | 0.52 | 30 | 7 | 4.839 | 3.5 | 0.821 |
|  | BG | 31 | 74 | 4.438 | 0.437 | 0.501 | 0.145 | 274.6 | **0.039** | 31 | 5 | 3.444 | 2.631 | 0.733 |
|  | CK | 25 | 62 | 3.888 | 0.379 | 0.456 | 0.173 | 316.1 | **0.04** | 26 | 4 | 1.38 | 1.145 | 0.286 |
|  | DB | 37 | 78 | 4.535 | 0.401 | 0.519 | 0.242 | 111.2 | 0.244 | 36 | 6 | 3.028 | 2.378 | 0.689 |
|  | HK | 25 | 72 | 4.5 | 0.409 | 0.524 | 0.281 | 52.7 | **0.005** | 31 | 4 | 2.458 | 2.119 | 0.613 |
|  | LS | 35 | 65 | 3.851 | 0.381 | 0.477 | 0.174 | 2000 | 0.221 | 35 | 4 | 1.267 | 0.867 | 0.217 |
|  | Pi | 29 | 73 | 4.476 | 0.401 | 0.506 | 0.168 | 319 | **0.03** | 30 | 4 | 3.061 | 2.328 | 0.697 |
|  | S | 29 | 60 | 3.631 | 0.432 | 0.434 | -0.017 | 2000 | 0.104 | 30 | 3 | 1.145 | 0.533 | 0.131 |
|  | SZ | 30 | 63 | 3.76 | 0.376 | 0.452 | 0.116 | 88.4 | **0.027** | 30 | 2 | 1.22 | 0.621 | 0.186 |
|  | TG | 26 | 74 | 4.553 | 0.423 | 0.509 | 0.161 | 243 | **0.028** | 30 | 6 | 3.689 | 3.042 | 0.754 |
|  | TMO | 31 | 79 | 4.727 | 0.417 | 0.539 | 0.201 | 35.1 | 0.105 | 30 | 5 | 3.041 | 2.471 | 0.694 |

| Allopatric reference *P. sylvestris* | CH | 30 | 90 | 5.37 | 0.498 | 0.574 | 0.163 | 111.8 | **0.006** | 30 | 4 | 3.383 | 2.475 | 0.729 |
| --- | --- | --- | --- | --- | --- | --- | --- | --- | --- | --- | --- | --- | --- | --- |
|  | DBS | 30 | 70 | 4.276 | 0.503 | 0.514 | 0.069 | 31.5 | 0.243 | 31 | 2 | 1.067 | 0.258 | 0.065 |
|  | NAR | 32 | 81 | 4.746 | 0.489 | 0.52 | 0.062 | 465.8 | **0.036** | 32 | 7 | 5.02 | 3.646 | 0.827 |
|  | PZ | 21 | 66 | 4.305 | 0.446 | 0.492 | 0.159 | 90.9 | **0.011** | 29 | 5 | 2.604 | 2.023 | 0.638 |
|  | RPN1 | 29 | 78 | 4.723 | 0.463 | 0.521 | 0.073 | 2000 | **0.034** | 28 | 4 | 2.562 | 2.008 | 0.632 |
|  | RPN2 | 24 | 82 | 5.01 | 0.476 | 0.494 | 0.019 | 2000 | **0** | 24 | 5 | 2.851 | 2.43 | 0.678 |
|  | RPN3 | 24 | 73 | 4.586 | 0.472 | 0.495 | 0.019 | 2000 | **0.009** | 30 | 6 | 2.273 | 2.286 | 0.579 |
|  | RPN4 | 22 | 77 | 4.913 | 0.482 | 0.535 | 0.12 | 34.5 | 0.252 | 22 | 6 | 3.408 | 2.83 | 0.74 |
|  | RPN5 | 30 | 91 | 5.218 | 0.486 | 0.531 | 0.129 | 98.6 | **0.006** | 25 | 6 | 3.205 | 2.708 | 0.717 |
|  | RPN6 | 25 | 75 | 4.625 | 0.44 | 0.515 | 0.139 | 2000 | **0.011** | 26 | 4 | 2.467 | 2.135 | 0.618 |
|  | RPN7 | 24 | 80 | 5.01 | 0.525 | 0.566 | 0.077 | 547.6 | 0.189 | 24 | 5 | 3.064 | 2.461 | 0.703 |
|  | RPN8 | 24 | 76 | 4.662 | 0.502 | 0.519 | 0.065 | 614 | **0.009** | 24 | 5 | 2.504 | 2.361 | 0.627 |
|  | S_B | 30 | 77 | 4.541 | 0.477 | 0.512 | 0.048 | 2000 | 0.495 | 30 | 5 | 2.961 | 2.339 | 0.685 |
|  | S_Bo | 36 | 82 | 4.571 | 0.472 | 0.496 | 0.035 | 2000 | **0.039** | 33 | 7 | 4.109 | 3.204 | 0.78 |
|  | S_By | 31 | 79 | 4.681 | 0.53 | 0.535 | 0 | 2000 | 0.107 | 31 | 7 | 3.546 | 2.802 | 0.742 |
|  | S_Ch | 32 | 86 | 4.762 | 0.487 | 0.512 | 0.038 | 2000 | 0.077 | 30 | 4 | 1.931 | 1.479 | 0.499 |
|  | S_Cz | 30 | 73 | 4.414 | 0.471 | 0.498 | 0.03 | 2000 | 0.1 | 30 | 6 | 1.948 | 2.041 | 0.503 |
|  | S_G | 32 | 88 | 4.907 | 0.493 | 0.502 | -0.002 | 2000 | **0** | 30 | 7 | 4.206 | 3.339 | 0.789 |
|  | S_Go | 31 | 82 | 4.842 | 0.544 | 0.536 | -0.024 | 248.6 | 0.079 | 31 | 6 | 2.445 | 2.006 | 0.611 |
|  | S_H | 30 | 76 | 4.54 | 0.485 | 0.513 | 0.038 | 685.6 | **0.028** | 29 | 7 | 3.738 | 3.019 | 0.759 |
|  | S_J | 30 | 78 | 4.619 | 0.507 | 0.518 | 0.01 | 2000 | **0.006** | 29 | 4 | 2.739 | 2.011 | 0.658 |
|  | S_K | 30 | 75 | 4.382 | 0.488 | 0.51 | 0.024 | 2000 | 0.272 | 30 | 5 | 2.885 | 2.323 | 0.676 |
|  | S_KB | 30 | 80 | 4.762 | 0.505 | 0.535 | 0.066 | 2000 | **0.042** | 30 | 8 | 3.191 | 2.94 | 0.71 |
|  | S_L | 30 | 76 | 4.48 | 0.479 | 0.511 | 0.043 | 155.9 | 0.329 | 30 | 5 | 2.903 | 2.413 | 0.678 |
|  | S_M | 32 | 80 | 4.622 | 0.52 | 0.518 | -0.025 | 2000 | **0.047** | 32 | 7 | 3.325 | 2.907 | 0.722 |
|  | S_Md | 33 | 80 | 4.673 | 0.504 | 0.525 | 0.039 | 499.7 | 0.086 | 30 | 3 | 1.411 | 1.09 | 0.301 |
|  | S_Mi | 31 | 77 | 4.486 | 0.491 | 0.504 | 0.015 | 1056.1 | 0.277 | 31 | 5 | 3.193 | 2.377 | 0.71 |
|  | S_MS | 31 | 80 | 4.608 | 0.44 | 0.486 | 0.114 | 344.9 | **0.025** | 30 | 7 | 3.516 | 3.116 | 0.74 |
|  | S_P | 32 | 77 | 4.614 | 0.504 | 0.519 | 0.013 | 2000 | 0.269 | 32 | 5 | 3.483 | 2.709 | 0.736 |
|  | S_Pi | 30 | 87 | 4.875 | 0.508 | 0.506 | -0.013 | 451.8 | **0.006** | 29 | 5 | 2.813 | 2.323 | 0.667 |
|  | S_Pl | 30 | 85 | 4.861 | 0.5 | 0.511 | 0.008 | 2000 | 0.099 | 30 | 8 | 3.147 | 2.765 | 0.706 |
|  | S_Po | 32 | 78 | 4.498 | 0.511 | 0.495 | -0.035 | 162.6 | 0.198 | 31 | 7 | 2.707 | 2.838 | 0.652 |
|  | S_R | 34 | 81 | 4.646 | 0.479 | 0.491 | 0.01 | 2000 | **0.034** | 32 | 6 | 2.723 | 2.179 | 0.653 |
|  | S_S | 31 | 82 | 4.661 | 0.491 | 0.493 | 0.01 | 574.8 | **0.047** | 31 | 7 | 2.886 | 2.461 | 0.675 |
|  | S_Su | 31 | 87 | 4.894 | 0.479 | 0.505 | 0.039 | 2000 | **0.001** | 31 | 5 | 3.28 | 2.649 | 0.718 |
| Allopatric reference *P. sylvestris* | S_We | 32 | 82 | 4.591 | 0.471 | 0.499 | 0.037 | 2000 | 0.08 | 32 | 6 | 2.142 | 2.131 | 0.55 |
|  | S_Wi | 30 | 76 | 4.518 | 0.505 | 0.514 | 0.046 | 51 | 0.269 | 30 | 6 | 2.813 | 2.257 | 0.667 |
|  | S_Wl | 30 | 84 | 4.84 | 0.45 | 0.509 | 0.107 | 450.1 | **0.006** | 28 | 6 | 2.882 | 2.343 | 0.677 |
|  | S_Wy | 30 | 79 | 4.566 | 0.462 | 0.485 | 0.032 | 2000 | **0.002** | 30 | 8 | 3.571 | 3.231 | 0.745 |
|  | So | 25 | 69 | 4.287 | 0.445 | 0.522 | 0.118 | 2000 | 0.594 | 40 | 1 | 1 | 0 | 0 |
|  | TAR | 15 | 50 | 3.846 | 0.462 | 0.472 | 0.006 | 68 | 0.513 | 20 | 4 | 1.695 | 1.607 | 0.432 |
|  | TLS | 26 | 63 | 3.983 | 0.423 | 0.499 | 0.182 | 39 | 0.466 | 30 | 4 | 2.239 | 1.525 | 0.572 |
|  | WGS | 32 | 85 | 4.875 | 0.463 | 0.517 | 0.111 | 65.1 | **0.041** | 32 | 5 | 4.031 | 2.869 | 0.776 |
|  | WK | 28 | 64 | 4.004 | 0.44 | 0.487 | 0.098 | 34.9 | 0.532 | 39 | 3 | 1.364 | 0.91 | 0.274 |
| Allopatric reference *P. uliginosa* | BAT | 27 | 68 | 4.12 | 0.42 | 0.486 | 0.113 | 2000 | **0.027** | 33 | 4 | 1.286 | 0.917 | 0.229 |
|  | WGB | 32 | 58 | 3.571 | 0.439 | 0.462 | 0.098 | 298.3 | 0.538 | 31 | 4 | 2.131 | 1.862 | 0.548 |
|  | WL | 19 | 65 | 4.411 | 0.457 | 0.526 | 0.111 | 268.1 | 0.396 | 25 | 4 | 2.306 | 1.954 | 0.59 |

N – number of samples; A – number of alleles; A_R_ – allelic richness; H_o_ – observed heterozygosity; H_e_ – expected heterozygosity; F – fixation index; N_E_ – effective population size (2000 = infinite); B. p. v – Bottleneck test p-value, significant results in bold; H_A_ – number of haplotypes; N_e_ – effective number of haplotypes; R_h_ – haplotype richness; H_e_ – haplotype variation
